# Effective degradation of zearalenone by multiple microbial isolates

**DOI:** 10.7717/peerj.20920

**Published:** 2026-04-03

**Authors:** Mbalenhle Ncongwane, Samkeliso Takaidza, Patience Chihomvu, Tharsius Raja William Raja, Michael Pillay

**Affiliations:** 1 Department of Natural Sciences, Vaal University of Technology, Vanderbijlpark, Gauteng, South Africa; 2 Department of Life and Consumer Sciences, College of Agriculture and Environmental Sciences, University of South Africa, Florida, Gauteng, South Africa

**Keywords:** Mycotoxins, Zearalenone (ZEN), Feed, Economic losses, Bacteria, De, Detoxify

## Abstract

Zearalenone (ZEN) is an estrogenic mycotoxin synthesized by specific fungal species that frequently colonize cereal crops such as maize, which is a fundamental component of animal feed. This mycotoxin induces significant reproductive disorders in swine and other livestock, resulting in considerable economic loss in the animal husbandry sector. ZEN contamination has emerged as a critical issue concerning global food and feed safety. The objective of this research was to isolate and characterize microorganisms from samples that can effectively detoxify ZEN. The samples were from six distinct sources: porcine feces, piggery soil, avian intestines, unpasteurized bovine milk, and feed that was left to mold. The spread plate technique was used to culture the samples and obtain pure cultures. The samples were identified by bacterial morphology and 16S rRNA gene sequencing. All the bacterial isolates identified in this study exhibited mycotoxin-degrading capabilities and included known ZEN-degrading species, *Bacillus amyloliquefaciens* and *B. subtilis*, with ZEN degradation ranging from 84.21% to 99.58%. A pure culture derived from piggery soil displayed particularly robust ZEN-degrading potential. Microscopic, biochemical, and molecular assessments substantiated the classification of this isolate as *Priestia megaterium*. This bacterium demonstrated ZEN-degrading ability (up to 0.5 mg/mL) and an elevated level of tolerance to various antibiotics (Multiple Antibiotic Resistance (MAR) index = 0.07). Additional research is necessary to investigate its potential application in the detoxification of ZEN.

## Introduction

Naturally occurring molds, particularly fungi from the genera *Fusarium* and *Gibberella* produce zearalenone (ZEN). Other notable species that synthesise ZEN include *Fusarium graminearum*, *F. culmorum*, *F. cerealis*, *F. equiseti*, *F. crookwellense*, and *F. semitectum* (Janik et al., 2020; Caglayan, Sahin & Ustundag, 2022). This mycotoxin is widely recognized for its prevalence in cereal crops, including maize, barley, wheat, and sorghum, as well as their derivatives (Khodaei, Javanmardi & Khaneghah, 2021; Chang et al., 2024). The widespread occurrence of ZEN presents considerable health hazards for both humans and animals (Rai, Das & Tripathi, 2020; Yu et al., 2024).

ZEN is categorized as a xenoestrogen, indicating that its chemical configuration closely mimics that of natural estrogens, thereby enabling it to disrupt hormonal equilibrium (Wang, Chen & Chen, 2021). This characteristic has been correlated with reproductive abnormalities in livestock, as well as hyperestrogenic syndromes in humans (Yu & Pedroso, 2023). In addition to its endocrine-disrupting properties, ZEN displays significant genotoxic and cytotoxic characteristics, which have been linked to the potential onset of cervical cancer and detrimental effects on essential organs such as the liver, spleen, kidneys, and reproductive system (Yang et al., 2022). The contamination of food and water represents an escalating concern, with research identifying ZEN and its metabolites in animal products such as milk (Murtaza et al., 2022), chicken eggs (Wang et al., 2024), and even potable water (Yu et al., 2024).

Despite advancements in agricultural methodologies, the complete eradication of ZEN-producing fungi continues to pose a significant challenge (Bryła et al., 2022). To reduce ZEN contamination, several physical and chemical techniques have been investigated. These consist of ozone gas treatments and the application of substances such as clays, activated charcoal, bentonite, cholestyramine, and 1,3-beta-D-glucan (Wu et al., 2021). However, these methodologies have limitations concerning efficacy, cost, and potential repercussions on food quality. Consequently, alternative approaches are necessary to diminish ZEN contamination in food and feed effectively.

Although numerous physical and chemical methodologies, including ozonation, adsorption using activated clays, and chemical binding agents, have been used to detoxify ZEN, these methods frequently encounter significant challenges, such as incomplete removal, elevated operational costs, and adverse effects on food palatability and nutritional quality (Wu et al., 2021). Conversely, biological detoxification presents a superior strategy, as it depends on specific microorganisms and their enzymes that selectively degrade ZEN under mild conditions while avoiding the production of harmful by-products. Microbial biodegradation is environmentally sustainable, economically viable, and preserves the nutritional integrity of feed, making it more advantageous than chemical treatments (Rogowska et al., 2022; Ntungwe, Tchana & Abia, 2024; Chang et al., 2025). A variety of microorganisms break down ZEN through enzymatic reduction, lactone ring cleavage, or hydrolysis producing metabolites such as *α*-zearalenol, *β*-zearalenol, or smaller non-estrogenic compounds (Rogowska et al., 2022; Gari & Abdella, 2023). Recent advancements in microbial biotechnology, including the genetic engineering of *Bacillus* and *Acinetobacter* strains, further augment the efficiency and stability of ZEN degradation, emphasizing biological detoxification as a safer and more practical alternative for large-scale applications (Zhou et al., 2023; Adeniji et al., 2025).

Biological degradation has emerged as a viable alternative owing to its high specificity, mild operational parameters, and minimal influence on nutrient composition (Ponnusami et al., 2023). The biological significance of these degradation products is attributed not only to the efficiency of detoxification but also to the stability and reduced estrogenic activity of the resulting compounds (Chang et al., 2025; Zhou et al., 2023). *Bacillus* species, including *B. licheniformis*, *B. subtilis*, and *B. amyloliquefaciens*, have been documented to convert ZEN into less toxic derivatives that exhibit stability under gastrointestinal conditions (Hsu et al., 2018; Wu et al., 2024). However, notwithstanding these advancements, a comprehensive evaluation of degradation pathways and product stability remains imperative in establishing the genuine detoxification potential of microbial strains. Furthermore, the influence of environmental factors such as pH, along with the potential adsorption of ZEN by bacterial isolates, must be taken into account to distinguish between degradation and physical binding. Consequently, further investigations should evaluate bacterial growth in the presence and absence of ZEN and monitor the temporal progression of ZEN reduction to ascertain whether the observed detoxification is attributable to true biodegradation.

The purpose of this study was to identify new microbes from selected samples that can degrade ZEN. The specific goals were (i) to isolate microorganisms that could degrade ZEN, (ii) to characterize the isolates phenotypically and biochemically, (iii) to use High-Performance Liquid Chromatography (HPLC) to evaluate ZEN degradation, (iv) to evaluate the antibiotic resistance profiles of the isolates, and (v) to use 16S rRNA gene amplification to identify the species that degrade ZEN.

## Materials and Methods

### Chemicals, reagents, and media

Zearalenone (ZEN) powder was purchased from Sigma-Aldrich (St. Louis, MO). Exactly 15.6 mg of ZEN powder was dissolved in 31.2 mL of acetonitrile (Glentham Life Sciences, Corsham, UK) to produce a stock solution with a concentration of 0.5 mg/mL. The stock solution was stored at −20 °C. The stock solution was diluted in methanol to prepare ZEN standard solutions for spiking experiments and calibration of High-Performance Liquid Chromatography (HPLC). Ultrapure water from a Direct-Q Millipore purification system was used to prepare the HPLC mobile phase. All additional reagents were purchased from Sigma-Aldrich and were of analytical quality.

### Sample selection

The six distinct sources, namely porcine feces, piggery soil, chicken gizzards, avian intestines, unpasteurized bovine milk, and moldy feed, were selected due to their representation of natural reservoirs of microorganisms frequently exposed to ZEN and other *Fusarium* mycotoxins. Pigs exhibit particular sensitivity to ZEN contamination, and both feces and piggery soil harbour diverse gut- and soil-associated microbes with the capability to degrade mycotoxins (Yang et al., 2021; Hein et al., 2024). Avian intestines were included, as poultry gut microbiota are known to encompass *Bacillus* and *Lysinibacillus* species that have been previously reported to degrade ZEN (Wang et al., 2018; Chang et al., 2025). Raw milk was chosen due to the potential presence of *Clostridium* and *Enterobacter* species in unpasteurized dairy products, which have been implicated in mycotoxin metabolism (Feligini et al., 2014; Murtaza et al., 2022). Moldy feed represents a niche wherein toxin-producing *Fusarium* coexists with competitive microbial communities, rendering it a rich source of potential ZEN-degrading isolates (Lee, Cheng & Liu, 2017; Harcarova et al., 2022). All of these sources were selected in order to increase the possibility of separating new, ecologically adapted bacteria with effective ZEN-degrading capabilities.

### Isolation of microorganisms

Isolation of microorganisms was performed with the use of the spread plate technique on porcine feces and piggery soil collected at Dreamland Piggery (26.7307°S, 27.7096°E), unpasteurized bovine milk that was purchased at an informal vendor, chicken intestines, chicken gizzards, and corn feed that had a 2-week moulding period. Sample collection was authorized verbally by Dreamland Piggery’s owner, Mrs. Anna Phosa (+27-814312441). Prof. Michael Pillay and Ms. Mbalenhle Ncongwane collected the samples on-site at the coordinates of 26.7307°S, 27.7096°. Non-selective nutrient agar (NA) was isolated to obtain a wide and non-selective culture of microorganisms. The aim of using this non-selective method was to prevent bias in neither enrichment of only highly competitive or ZEN-resistant bacteria nor to permit the future screening of functions to determine which individual isolates were actually ZEN-degrading. Though the isolation process can be simplified by using ZEN-selective media, which helps to enrich ZEN-tolerant bacteria, alternative methods might not detect organisms that degrade ZEN in a cometabolismic manner or express degradation activity in non-stressful and nutrient-rich conditions. To isolate, every sample (1 g/1 mL) was suspended in nine mL of phosphate-buffered saline (PBS), vortexed and left to rotate for about 30 min before plating 100 µL of each dilution on NA plates and incubating at 37 °C for 24 h. Pure isolates were obtained by morphologically different colonies subcultured, and were stored in 50% sterile glycerol at −80 °C. Two fungal isolates were selected using this method including two mouldy corn fungi, and 63 morphologically distinct bacterial isolates.

### Phenotypic characteristics of the isolates

The isolates were cultured on nutrient agar plates at 37 °C for 24 h, and the colony morphology was observed. Biochemical tests were carried out according to methods described in Bergey’s Manual of Determinative Bacteriology (1923).

### Screening of ZEN-degradation ability of the isolates

The capability of bacterial and fungal isolates to degrade Zearalenone (ZEN) was systematically evaluated in Luria-Bertani (LB) broth and Potato Dextrose Broth (PDB), respectively. LB broth (Sigma-Aldrich, St. Louis, MO, USA) and PDB (Merck, Darmstadt, Germany) were produced according to the manufacturers’ requirements. Before sterilisation, the initial pH of both LB and PDB medium was raised to 7.0 using either 1 M NaOH or 1 M HCl. A calibrated pH meter (Mettler Toledo) was used to measure the pH both before and after incubation. After a 24-hour incubation period, no obvious pH drift was found, suggesting that the broth stayed steady at pH 7 during the degradation test. In each assay, 10 µL of a ZEN standard solution (3.5 µg/mL) was introduced into 990 µL of microbial culture and incubated at 37 °C for 24 h. The final concentration of ZEN in the bacterial cultures was 25 µg/mL. Control experiments consisted of sterile media devoid of microbial presence. After incubation, methanol was added, followed by vortexing and centrifugation at 12,000 rpm for 10 min. The supernatant was filtered (0.22 µm) before HPLC analysis. In total, 63 bacterial isolates were screened for their ZEN-degradation ability, together with 2 fungal isolates. Methanol was used to generate ZEN standards with concentrations ranging from 0.5 mg/mL to 3.5 µg/mL. HPLC analysis was performed utilizing the Agilent 1260 Infinity Quaternary system, which is equipped with a Diode-Array Detector, a quaternary isocratic pump, and an auto-injector. A C18 reversed-phase column (250 × 4 mm, 5 µm) was filled with a 20 µL sample and kept at 20 ± 0.8 °C. The mobile phase, consisting of methanol, water, and acetonitrile in the ratio of 22:43:35 (v/v/v), was filtered (0.45 µm) and degassed before use. A wavelength of 235 nm (with a reference of four nm) and a flow rate of 1.0 mL/min were used for the detection. Linearity was validated using ZEN standards ranging from 0.5 to 3.5 µg/mL, and specified the volume of methanol added (1:1 v/v to the culture supernatant). The concentrations were determined by correlating sample peak areas with those of the standards. The percentage of ZEN degraded was determined using the following formula:

\begin{eqnarray*}\text{The percentage of ZEN degradation}= \left\{ 1- \left[ \frac{ZEN~peak~area~of~sample}{ZEN~peak~area~of~standard} \right] \right\} \times 100. \end{eqnarray*}

Isolates that showed degradation capabilities were identified using molecular methods.

### Determination of antibiotic resistance

The disc diffusion methodology described by Patel et al. (2011) was used to assess the sensitivity of the 16 bacterial isolates to a range of antibiotics. The antibiotics encompassed Ampicillin (10 µg), Amoxicillin (25 µg), Augmentin (30 µg), Azithromycin (15 µg), Cephalexin (30 µg), Cephalothin (30 µg), Clinafloxacin (5 µg), Ciprofloxacin (5 µg), Colistin sulfate (10 µg), Cotrimoxazole (25 µg), Gentamicin (10 µg), Nitrofurantoin (300 µg), Sulphatriad (300 µg), Nalidixic acid (30 µg), Tetracycline (30 µg), Streptomycin (10 µg), and Trimethoprim (5 µg). Three isolates (PI3, PI4, and CI4) were subjected to evaluation against the Mastring M51 panel; however, the predominant number of isolates (13 out of 16) were analyzed utilizing the Mastring M14 panel. Given that isolate CI5 did not proliferate when assessed with M14 antibiotics, it was subsequently tested only against bacteria that exhibited strong ZEN degradation ability. A sterile cotton swab was employed to evenly distribute 100 µL of bacterial culture onto Mueller-Hinton agar for each antibiotic. Antibiotic discs were strategically placed upon the agar surface following a ten-minute absorption period of the culture by the medium. The plates were incubated for twenty-four hours at 37 °C. Isolates were classified as sensitive if the diameter of the inhibition zone was 12 mm or greater, with zones of inhibition measured in millimeters (mm) (Patel et al., 2011).

### Molecular identification of the isolates

#### CTAB DNA extraction method

The genomic DNA of 16 bacterial and one fungal isolate that showed ZEN degradation was isolated using the CTAB method with slight modifications. Exactly, 700 µL of cell culture was centrifuged at 12,000 rpm for 15 min at 4 °C. The supernatant was discarded, and 700 µL of CTAB lysis buffer was added to the pellet. The mixture was vortexed and incubated at 65 °C for 20 min in a water bath. After that, the samples were centrifuged at 10,000 rpm for 10 min. The supernatant was transferred to a clean tube, and 700 µL chloroform isoamyl alcohol 24:1 was added. The mixture was vortexed to mix well and centrifuged at 10,000 rpm for 10 min. After centrifugation, the aqueous upper layer was transferred to a new sterile tube, ensuring the bottom layers were not disturbed. Then 600 µL ice-cold ethanol and 150 µL 5 M NaCl solution were added. The solution was mixed by inversion and centrifuged at 13,000 rpm for 10 min. The supernatant was discarded, and the pellet was washed with 600 µL of 70% ethanol. After adding ethanol, the solution was mixed and centrifuged at 10,000 rpm for 5 min. The ethanol was discarded, and the pellet was allowed to air dry for 30 min. Exactly 50 µL of TE buffer was added to the pellet and re-suspended. The concentration of DNA was then checked using nanodrop, and DNA was stored at 4 °C for further analysis.

#### Polymerase chain reaction

The partial 16S rRNA gene sequences of 17 isolates were amplified by Polymerase Chain Reaction (PCR) using universal 16S rRNA gene primers 16S-27f (5′AGAGTTTGATCCTGGCTCAG3′) and 16S-1492r (5′GGTTACCTTGTTACGACTT3′) (Weisburg et al., 1991). Twenty-five µL of Emerald master mix, 2.5 µL of each primer, 11 µL of distilled water, and 9 µL of template DNA (1.99–2.05 µg/mL) were all included in each 50 µL PCR reaction. To confirm that there was no contamination, a negative control that contained all the reagents except DNA was added. An Eppendorf 96-well Thermocycler was used for PCR amplification, and the cycling conditions were as follows: 5-minute initial denaturation at 95 °C, 29 cycles of denaturation at 94 °C for 1 min, annealing at 46.5 °C for 1 min, extension at 72 °C for 1 min and 30 s, and a final extension at 72 °C for 10 min. Following separation on a 2.0% agarose gel, the PCR products were stained with 5 µL of ethidium bromide (1 mg/mL stock) and examined under a UV lamp. For sequencing, PCR products were shipped to Inqaba Biotech in Pretoria, South Africa. The resulting sequences were compared to those published in the GenBank database accessed through the NCBI website (http://www.ncbi.nlm.nih.gov) using the BLAST method.

### Phylogenetic tree analysis

Phylogenetic relationships were deduced from the 16S rRNA gene sequences of the bacterial isolates. The closest related species were selected for study after the acquired sequences were compared with reference sequences obtained from the NCBI GenBank database. MEGA X software’s Neighbour-Joining (NJ) method was used to build the phylogenetic tree. To illustrate the genetic difference between taxa, evolutionary distances were computed and branch lengths are displayed in the tree. The isolates were grouped with reference species from Bacillus (*B. subtilis*, *B. amyloliquefaciens*, *Priestia megaterium*, *Sporosarcina* sp.), Enterobacter (mostly *E. hormaechei*), and *Acinetobacter* spp. based on clustering characteristics. The closest phylogenetic grouping with verified reference sequences was used to determine species-level connections.

## Results

The samples used in this study included pig manure, soil from the same piggery, the caecal content of chicken intestine, unpasteurized cow’s milk, and moldy corn feed incubated for two weeks in a dark cupboard. The extensive use of maize as a main ingredient in animal feed and the high susceptibility of pigs to ZEN contamination led to the selection of these sample sources.

### Culture and biochemical tests of the samples

**Figure 1:**
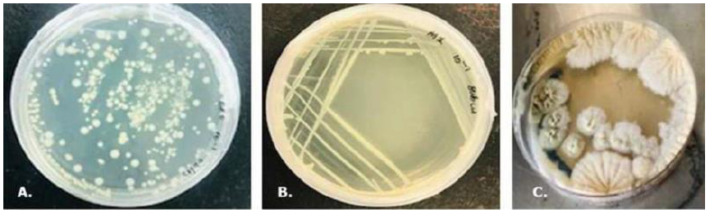
Macroscopic morphology of some of the isolates from the different samples. (A) CI1 10^−5^ (from the chicken intestines), (B) M1 10^−1^ (from the milk), (C) MC1 10^−3^ (from the mouldy corn). To streamline identification and eliminate redundant isolates, Gram staining, endospore staining, and acid-fast staining were performed. Biochemical characterization was subsequently carried out on the distinct isolates to assess their potential for further investigation.

Table 1 provides an overview of the morphological classification of the strains that were isolated from various sources. The morphological characterization encompassed colony form, elevation, edge, pigmentation, and texture. Phenological traits were also meticulously documented, including growth rate, sporulation, and colony development under diverse incubation durations and nutrient conditions. Biochemical assays were conducted to evaluate catalase, oxidase, starch hydrolysis, carbohydrate fermentation, and nitrate reduction capabilities, among other factors. Collectively, these comprehensive observations facilitated the preliminary classification of the isolates before molecular validation, adhering to established methodologies in microbial taxonomy (Yang et al., 2021; Hein et al., 2024; Adeniji et al., 2025). This integrated dataset furnishes a robust foundation for correlating microbial diversity with ZEN-degrading potential. Colonies exhibiting distinct morphological characteristics such as variations in form, elevation, edge, color, and texture were selected for further study, as shown in Fig. 1. The cells were stained with a Gram Staining kit (Sigma-Aldrich, St. Louis, MO, USA) according to the manufacturer’s instructions, and then observed under a microscope. The cells were also stained for acid-fast properties and endospore formation. The biochemical assays employed for isolate preliminary identification before these phenotypic data supplemented molecular characterisation. A total of 130 microbial isolates were initially found. Morphologically distinct isolates were chosen for additional characterisation. Thus, only 63 microbial isolates underwent thorough morphological (Appendices SI and SII) and biochemical characteristics (Tables 2A and 2B).

**Table 1:** Morphological characteristics of bacterial isolates.

**Isolate code**	**Source**	**Gram reaction**	**Cell morphology**	**Arrangement**	**Colony colour**	**Colony surface**
P2 10^−1^	Pig faeces	+	Bacilli	Clusters, Streptobacillus	White/Cream	Smooth, Glistening
P1 10^−2^	Pig faeces	+	Bacilli	Clusters	White/Cream	Smooth, Glistening
P2 10^−3^	Pig faeces	+	Bacilli	Clusters	White/Cream	Smooth, glistening
P1 10^−4^	Pig faeces	+	Bacilli	Singular	White	Smooth
P1 10^−5^	Pig faeces	+	Bacilli	Clusters	White	Smooth
P3 10^−5^	Pig faeces	+	Bacilli	Singular	White	Smooth
P2 10^−6^	Pig faeces	+	Bacilli	Clusters	White	Smooth
P2 10^−7^	Pig faeces	+	Bacilli	Singular	White	Smooth
S1 10^−1^	Soil	+	Bacilli	Singular	White	Smooth
S4 10^−1^	Soil	+	Bacilli	Singular	White	Smooth
S3 10^−2^	Soil	+	Bacilli	Clusters	White	Smooth
S1 10^−3^	Soil	+	Bacilli	Clusters	White	Smooth
S3 10^−3^	Soil	+	Bacilli	Chains	White	Smooth
CI1 10^−1^	Chicken intestine	**+**	Bacilli	Singular	White	Smooth
CI2 10^−1^	Chicken intestine	–	Bacilli	Singular	White	Smooth
CI3 10^−1^	Chicken intestine	–	Bacilli	Singular	White	Smooth
CI2 10^−3^	Chicken intestine	–	Bacilli	Singular	White	Smooth
CI1 10^−7^	Chicken intestine	–	Bacilli	Singular	White	Smooth
G1 10^−1^	Chicken gizzard	–	Bacilli	Singular	White	Smooth
G1 10^−3^	Chicken gizzard	–	Bacilli	Singular	White	Smooth
M1 10^−1^	Milk	+	Bacilli	Singular	White	Smooth
M1 10^−4^	Milk	–	Bacilli	Singular	White	Smooth
M1 10^−7^	Milk	–	Bacilli	Singular	White	Smooth

Chicken gut samples contained *Vibrio* sp., *Proteus/Yersinia* sp., *Aeromonas* sp., *Clostridium* sp., *Neisseria* sp., *Klebsiella pneumoniae*, and *Enterobacter intermedius*, echoing findings from other poultry microbiome studies (Shang et al., 2018; Aruwa et al., 2021). Milk samples had *Clostridium* sp., *K. pneumoniae*, and *Proteus/Yersinia* sp., like isolates from small-scale dairies in Zimbabwe and Italy. While biochemical methodologies yielded preliminary identification of the isolates, 16S rRNA gene sequencing was selectively executed for *Bacillus* and *Enterobacter* species. Molecular validation ensured precise species-level identification of these functionally significant isolates.

### Analysis of ZEN degradation by the isolates

**Figure 2:**
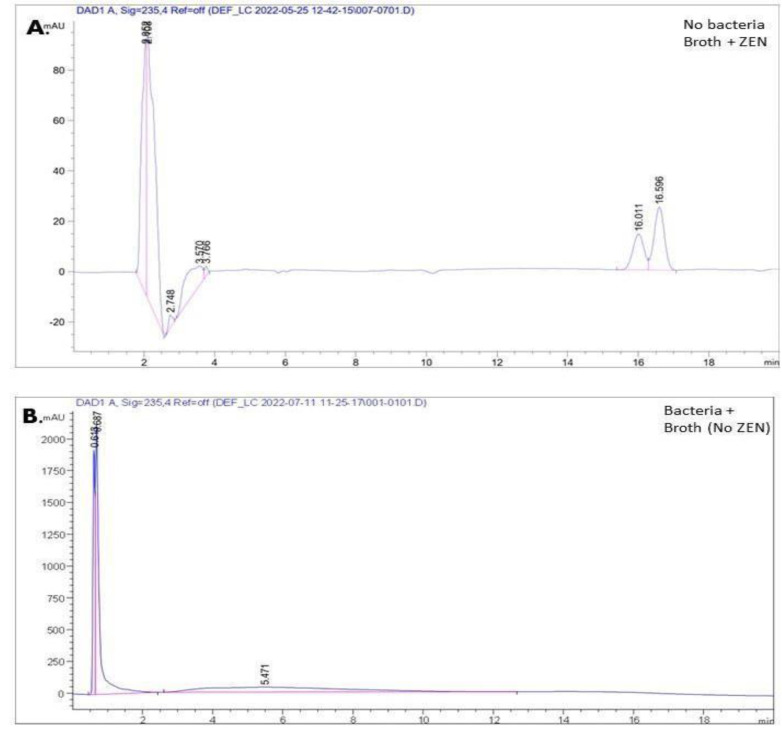
(A) ZEN control and (B) bacterial only control. Exactly 3.5 µg/mL of ZEN was added to the control (A). Sample B did not contain ZEN.

HPLC was used to determine the ZEN degradation by the isolates. A standard curve was created to assess the linearity of the procedure, with doses ranging from 0.5 to 3.5 µg/mL. A regression coefficient (R^2^) of ≥ 0.9 confirmed the reliability of the regression model. This validated HPLC procedure is suitable for the quantitative analysis of residual ZEN after degradation experiments. Accuracy was evaluated by comparing the measured ZEN concentrations with the known values. HPLC analysis revealed two peaks around 16 min for ZEN (Fig. 2A). HPLC analysis of the ZEN standard disclosed two closely eluting peaks at approximately 16 min, which aligns with the presence of cis- and trans-isomers of ZEN known to manifest under UV-Vis irradiation and chromatographic conditions (De Saeger, Sibanda & Van Peteghem, 2003; Vo et al., 2024). These isomeric forms represent degradation-related structural variants of ZEN rather than distinct and unrelated metabolites. Prior research has established that the enzymatic degradation of ZEN frequently produces such stereoisomers or modified intermediates. In the present investigation, the elimination of both peaks after bacterial incubation confirmed the complete degradation of ZEN by the isolates. The absence of peaks at the expected retention time (Fig. 2B) confirmed the absence of ZEN in the sample. ZEN degradation efficiency ranged from 84.21% to 99.58% for the isolates. Antibiotic susceptibility of ZEN-degrading isolates determined by the disc diffusion assay is shown in Appendix SIII. Appendix SIV shows the comprehensive ZEN degradation rate calculations for the 17 isolates. Their overall degradation efficiency is summarized as a percentage in Table 3. There was no noticeable degradation in the control (ZEN in sterile LB medium without bacterial inoculation), indicating that all reductions were caused by microbial action. The range of the degradation rates was 84.21% to 99.58%. Of the isolates, CI2 had the lowest degradation rate (84.21%), while SO1 (from soil) had the highest (99.58%). The isolates were classified based on their degradation performance based on the % reduction of residual ZEN ascertained by HPLC analysis. Isolates with ZEN degradation levels between 90% and 98% were characterized as moderate degraders, while isolates with degradation levels more than 98% were classified as strong degraders. This classification system aligns with previously documented standards used in ZEN biodegradation research, including HPLC measurements and microbiological screening (Hsu et al., 2018; Lee, Cheng & Liu, 2017; Wu et al., 2024). As a result, isolates CI3, CI5, CC2, MI1, MI2, MI3, PI2, PI3, and PI4 were found to be strong ZEN degraders (>98%), whereas isolates CI4, CC1, MC1, PI1, and SO3 were categorised as intermediate degraders (90–98%).

**Table 2:** Summary of morphological classification.

**(A) Biochemical tests on the Gram-positive microbes**									
**Organism**	**SF**	**AF**	**SA**	**C**	**SH**	**VP**	**M**	**NR**	**Bacterial sp.**
P2 10^−1^	–	–		+	–				*Corynebacterium xerosis*
P1 10^−2^	–	–		+	–				*C. xerosis*
P2 10^−3^	–	–		+	–				*C. xerosis*
P1 10^−4^	+		–	+	–			–	*Bacillus sphaericus*
P1 10^−5^	–	–		+	–				*C. xerosis*
P3 10^−5^	+		–	+	–			+	*B. pasteurii*
P2 10^−6^	–	–			–				*C. xerosis*
P2 10^−7^	+		–	+	–			–	*B. sphaericus*
S1 10^−1^	+		–	+	–			–	*B. sphaericus*
S4 10^−1^	+		–	+	–			–	*B. sphaericus*
S3 10^−2^	–	–		+	+				*C. kutsceri*
S1 10^−3^	–	–		+	+				*C. kutsceri*
S3 10^−3^	+		–		+	+	–		*B. anthracis*
CI1 10^−1^	+		+						*Clostridium* sp.
M1 10^−1^	+		+						*Clostridium* sp.

**(B) Biochemical tests for the Gram-negative microbes**											
**Organism**	**O**	**GF**	**LF**	**NG**	**VP**	**I**	**MR-VP**	**U**	**H**_**2**_ **S P**	**M**	**Bacterial sp.**
G1 10^−1^	+	+		+							*Vibrio* sp.
G1 10^−3^	–		+			–	+/+				*Enterobacter intermedius*
CI2 10^−1^	+	+		+							*Vibrio* sp.
CI3 10^−1^	–		–			–		+			*Proteus/Yersinia* sp.
CI2 10^−3^	+	+		–	–						*Aeromonas* sp.
CI1 10^−7^	–		+			–	+/-		–	–	*Klebsiella pneumonia*
M1 10^−4^	–		–			–		+			*Proteus/Yersinia* sp.
M1 10^−7^	–		+			–	+/-		–	–	*Klebsiella pneumonia*

**Notes.**

SF Spore Forming AF Acid Fast SA Strict Anaerobe C Catalase SH Starch Hydrolysis VP Voges-Proskauer M Motility NR Nitrate Reduction + Positive - Negative O Oxidase GF Glucose Fermentation LF Lactose Fermentation NG NaCl Growth requirement VP Voges-Proskauer I Indole MR-VP Methyl Red- Voges-Proskauer U Urease H_2_S P Hydrogen Sulfide Production M Motility

**Figure 3:**
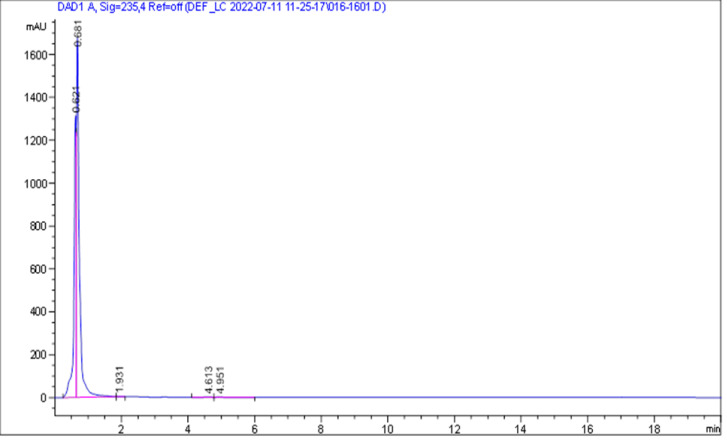
HPLC chromatogram of the soil sample (SO1) with 3.5 ppm of zearalenone. HPLC chromatogram of the soil sample (SO1) with 3.5 ppm of zearalenone HPLC chromatogram of soil isolate SO1 with 3.5 μg/mL of zearalenone.

According to the HPLC analysis, fourteen of the seventeen isolates exhibited high ZEN degradation (>95%), with three showing intermediate degradation (<91%) and one isolate (SO2) unable to degrade ZEN. The highest degradation rate, 99.58%, was achieved by a soil isolate. These isolates demonstrated efficient ZEN degradation at a starting concentration of 3.5 µg/mL. The soil isolate SO1, identified as *Priestia megaterium*, showed the most effective ZEN degradation (99.58%) (Fig. 3), at the initial ZEN concentration of 3.5 µg/mL, and demonstrated sensitivity to all tested antibiotics except ampicillin. One-way ANOVA revealed statistically significant variation among the ZEN degradation abilities of the isolates (*F* = 19.47, *p* <  0.001). Tukey’s HSD *post-hoc* test showed that SO1, PI4, CI5, MI1, MI2, and PI3 formed a significantly higher-performing group (*p* < 0.05) compared to moderate-performing isolates such as CI2 and CI1. These statistical results confirm that the observed differences in degradation efficiency were not random but reflect true functional variability among the isolates.

**Table 3:** Total ZEN degradation rates for the bacterial isolates.

**Isolates**	**Degradation rate (%)**
PI1	96.19
PI2	98.67
PI3	99.31
PI4	99.52
SO1	99.58
SO3	96.92
CC1	96.97
CC2	99.40
CI1	86.09
CI2	84.21
CI3	98.28
CI4	90.89
CI5	99.44
MI1	99.36
MI2	98.54
MI3	99.03
MC1	97.72

**Notes.**

The values are derived from three separate studies and show the mean % degradation ± standard deviation. Tukey’s *post hoc* test (*p* < 0.05) was used after one-way ANOVA to examine differences between isolates.

### Molecular identification of the best ZEN-degrading isolates

**Figure 4:**
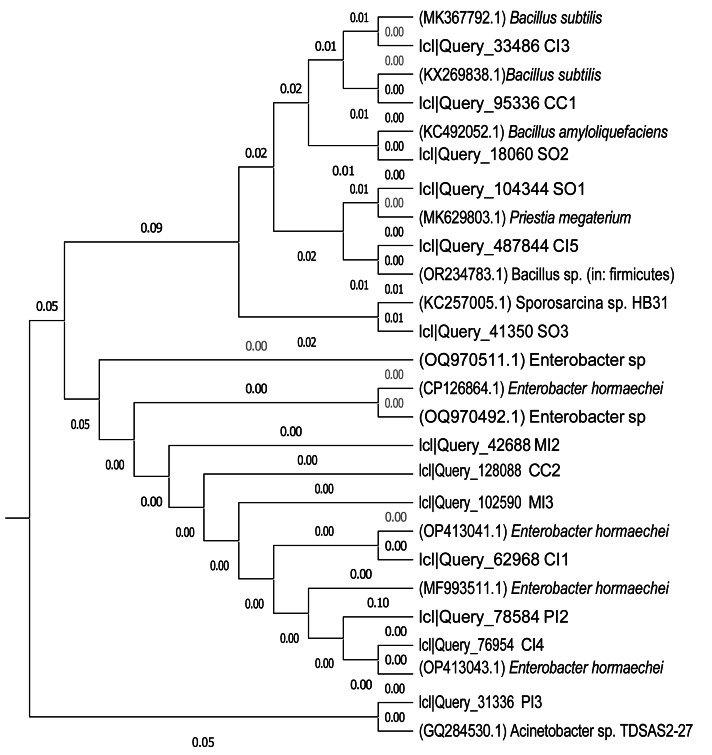
Cluster analysis showing the relationship among some isolates of this study and related bacterial species from BLAST analysis. The tree was constructed using the neighbour-joining method.

Clustering of the 13 bacterial isolates (accession numbers: PX057263, PX057264, PX057265, PX057266, PX057267, PX057268, PX057269, PX057270, PX057271, PX057272) was assessed using the 16S rRNA sequences and the neighbor-joining technique (Fig. 4). The results revealed that six isolates (CC2, CI1, CI4, MI2, MI3, and PI2) were closely related to *Enterobacter* species, while four isolates (CI3, CC1, SO2, and CI5) were linked to *Bacillus* species. Additionally, *Acinetobacter* sp., *Priestia megaterium*, and *Sporosarcina* sp. were associated with isolates PI3, SO1, and SO3, respectively. Notably, SO1, the isolate with the highest ZEN degradation efficiency, was closely related to *Priestia megaterium*.

## Discussion

Prior studies have shown that it is possible to successfully extract ZEN-degrading bacteria from comparable settings. For example, a soil sample containing significant ZEN-degrading capacity was used by Harcarova et al. (2022) to identify *Bacillus licheniformis* strain CK1. Furthermore, the isolation of *Lysinibacillus* sp. from the large intestine of chickens that demonstrated effective ZEN degradation was reported by Wang et al. (2018). Chang et al. (2025) isolated *B. mojavensis* with remarkable ZEN-degrading ability from the caecal content of hens. Additionally, Lee, Cheng & Liu (2017) showed that after 36 h of incubation, *B. amyloliquefaciens* LN, which was isolated from moldy maize, was able to degrade 92% of ZEN. These results demonstrate that microbial sources to reduce ZEN contamination have been isolated from similar samples used in this study.

The coexistence of cis and trans isomers, which are known to occur because of the structural flexibility of the ZEN molecule during chromatographic separation, may be the cause of the appearance of two peaks for ZEN in the HPLC chromatogram (De Saeger, Sibanda & Van Peteghem, 2003). Analytical investigations using reversed-phase HPLC have also reported this finding, showing that both geometric isomers are partially resolved under particular solvent compositions and column circumstances (Caglayan, Sahin & Ustundag, 2022). The current investigation used diode-array detection (DAD) at 235 nm to enable simultaneous detection of ZEN and its potential degradation products, even though fluorescence detection is frequently used for mycotoxin quantification due to its superior sensitivity and specificity (De Saeger, Sibanda & Van Peteghem, 2003). This method guarantees precise residual ZEN concentration monitoring free from microbial metabolite influence. Recent research examining ZEN degradation and microbial detoxification has effectively employed similar DAD-based techniques (Murtaza et al., 2022; Gari & Abdella, 2023). Consequently, the chromatographic and detection settings used in this investigation are appropriate for assessing how well the bacterial isolates degrade ZEN.

ZEN degradation rates were influenced by several factors, including the initial ZEN concentration (Gari & Abdella, 2023). Compared to other primary screening studies (Wu et al., 2024), these isolates exhibited superior ZEN degradation capabilities. For instance, *B. licheniformis* CK1 degraded 95.8% of ZEN (2 µg/mL) in 36 h (Hsu et al., 2018), while *Lysinibacillus* sp. ZJ-2016-1 achieved an 86.1% degradation rate. These findings highlight the potential of these microbial isolates for bioremediation and ZEN detoxification. The degradation potential of ZEN by microbial isolates is significantly modulated by environmental factors such as pH, culture medium, and incubation duration. Optimal degradation predominantly occurs at near-neutral pH, as extreme acidic or alkaline conditions may denature the microbial enzymes responsible for biodegradation (Gari & Abdella, 2023). The availability of nutrients within the culture medium also plays a pivotal role, as nutrient-rich media such as LB broth enhance microbial growth and enzymatic activity, thereby facilitating superior degradation efficiency in comparison to minimal media (Chang et al., 2025). Moreover, the incubation period directly influences degradation dynamics. The numerous studies indicate that ZEN degradation escalates within the initial 24–48 h, after which the rate stabilizes, likely attributable to diminished enzymatic activity (Lee, Cheng & Liu, 2017; Wu et al., 2024). In the current study, *Priestia megaterium* exhibited the most efficient degradation at pH 7 and after 24 h of incubation, underscoring the significance of optimizing these parameters for effective bioremediation.

The biochemical similarities between *Corynebacterium kutscheri* and *Priestia megaterium*, particularly in starch hydrolysis and catalase production, suggest a potential link between their enzymatic properties and ZEN degradation. The bacterial species identified in this study have all been linked to mycotoxin degradation, including ZEN. *Bacillus* species like *B. pumilus*, *B. amyloliquefaciens*, and *B. subtilis* are known to produce antifungal compounds such as lipopeptides, proteases, and *α*-amylases, which help suppress mycotoxin-producing fungi (Soliman, Khaleil & Metwally, 2022). Two isolates closely related to *B. subtilis* in this study demonstrated over 90% ZEN degradation, consistent with previous findings showing *B. subtilis* can detoxify both aflatoxin B1 and ZEN simultaneously (Wu et al., 2024).

The presence of *Enterobacter* species, commonly found in pigs, supports a previous report of these bacteria in pig gut flora (Hein et al., 2024). Furthermore, *Acinetobacter* sp., shown to degrade ZEN through extracellular extracts (Zhou et al., 2023), and *Priestia megaterium*, which has not been previously linked to ZEN degradation, were also identified. The high ZEN degradation rate (99.58%) demonstrated by SO1, identified as *P. megaterium* SO1, highlights its potential as a bioremediation agent. Given the recent taxonomic reclassification of *B. megaterium* to *Priestia megaterium* (Adeniji et al., 2025), further research is necessary to fully understand its role in mycotoxin breakdown and its potential applications in controlling toxigenic fungal growth.

The foremost ZEN-degrading strains delineated in the current investigation, *Priestia megaterium* (SO1), *Bacillus subtilis* (CI3), and *Enterobacter* sp. (CI1), have been systematically compared. *P. megaterium* demonstrated degradation that is likely facilitated through adsorption and enzymatic hydrolysis, as evidenced by the swift reduction of ZEN with no subsequent increase in degradation during extended incubation, in accordance with prior findings regarding *Bacillus*-related adsorption and enzymatic decomposition (Wu et al., 2024; Adeniji et al., 2025). *B. subtilis* is recognized for its secretion of lipopeptides, esterases, and oxidoreductases, which promote both adsorption and oxidative cleavage of ZEN into less toxic metabolites (Soliman, Khaleil & Metwally, 2022; Wu et al., 2024). In contrast, *Enterobacter* sp. has been associated with oxidoreductive biotransformation, converting ZEN into *α*- and *β*-zearalenol derivatives through reductase activity (Hein et al., 2024; Zhou et al., 2023). These mechanistic distinctions underscore that adsorption and enzymatic hydrolysis predominate in *P. megaterium*, whereas oxidative/reductive enzymatic pathways are more salient in *Bacillus* and *Enterobacter* spp. The comparative analysis indicates that the enhanced efficiency of *P. megaterium* is attributable to its dual mechanism of adsorption and degradation, rendering it the most promising strain for bioremediation endeavours.

## Conclusion

Many investigations have revealed that zearalenone (ZEN) can be broken down by microorganisms present in soil, rumen fluid, animal excrement, and the environment. The microbial degradation of ZEN is accomplished through a variety of enzymatic and metabolic mechanisms that differ among bacterial strains. Prior research has substantiated that *Bacillus* spp. use lactonase- and esterase-mediated hydrolysis of the lactone ring, resulting in less toxic products such as *α*-zearalenol and non-estrogenic derivatives. *Enterobacter* and *Acinetobacter* species primarily degrade ZEN through oxidoreductase-mediated pathways, modifying the hydroxylation pattern of the aromatic ring. *Priestia megaterium*, identified in this study as the most efficacious strain, exhibited rapid degradation (99.58%) under the experimental culture conditions employed, which may involve a synergistic mechanism of adsorption to cell surface polysaccharides and enzymatic hydrolysis. The occurrence of degradation without a concomitant effect on bacterial growth suggests that ZEN biotransformation is likely associated with constitutive metabolic enzymes rather than stress-induced detoxification. Therefore, our findings not only accentuate the superior degradation potential of *P. megaterium* but also establish it as a promising candidate for the bioremediation of ZEN-contaminated feed and food systems, necessitating further investigations into the specific enzymatic pathways involved.

One of the key findings of the study is that all of the bacteria were isolated based on their ability to degrade ZEN, and the selected strains demonstrated a strong ability. The observed peak degradation happened at pH 7 and 37 °C after 24 h, which were the default culture conditions employed for all isolates, even though the experimental design did not involve a full factorial optimisation of temperature, pH, or incubation period. Therefore, instead of being called optimal parameters, these conditions are called operational parameters. For this investigation, these parameters are considered operationally ideal due to their consistent pattern among isolates. However, further optimisation tests are still needed to verify that these circumstances are generally ideal for ZEN degradation. The soil isolate SO1 identified as *P. megaterium* was the most effective bacterium in breaking down ZEN. The current trials did not evaluate pH optimization or adsorption mechanisms. It is yet to be confirmed ZEN’s possible impact on bacterial proliferation. To clearly identify a connection between ZEN degradation and bacterial metabolism, future research should evaluate the growth of bacteria in the presence and absence of ZEN and track growth dynamics and ZEN reduction over time. According to these results, *P. megaterium* shows promise as a possible ZEN bioremediation agent; nevertheless, more investigation is required to clarify the underlying mechanisms and maximize its usefulness in reducing mycotoxin contamination in food and feed.

##  Supplemental Information

10.7717/peerj.20920/supp-1 Supplemental Information 1 Appendixes I, II, and III The supplemental file contains all of the datasets, including the microscopic appearance, colony morphology, and ZEN-degradation rate calculations of microbial isolates from a variety of sources (soil, raw milk, moldy maize, chicken intestine, gizzard, and piggery).

10.7717/peerj.20920/supp-2 Supplemental Information 2 Raw data

10.7717/peerj.20920/supp-3 Supplemental Information 3 Sequence data

## Additional Information and Declarations

### Field Study Permissions

The following information was supplied relating to field study approvals (i.e., approving body and any reference numbers): The pig feces and soil samples were obtained with permission from Dreamland Piggery (26.7307°S, 27.7096°E)

### Data Availability

The following information was supplied regarding data availability: The raw data are available in the Supplementary File. The sequences are available at GenBank: PX057271, PX057271, PX057267, PX057266, PX057270.
