# Supplementary material for: Effective degradation of zearalenone by multiple microbial isolates
Source: PeerJ. 2026 Apr 3;14:e20920. doi: 10.7717/peerj.20920 (PMC13052100; doi:10.7717/peerj.20920)
Supplement: Supplemental Information 1 — The supplemental file contains all of the datasets, including the microscopic appearance, colony morphology, and ZEN-degradation rate calculations of microbial isolates from a variety of sources (soil, raw milk, moldy maize, chicken intestine, gizzard, and piggery). [file peerj-14-20920-s001.docx]

**Supplementary Document**

# APPENDICES

**APPENDIX I**

**Colony morphology from the piggery sub-cultures**

| **Colony no.** | **Dilution factor** | **Form** | **Elevation** | **Margin** | **Colour** | **Surface appearance** | **Organism label** |
| --- | --- | --- | --- | --- | --- | --- | --- |
| 1 | 10^-1^ | Circular | Flat | Entire | C.W | Smooth | P1 10^-1^ |
| 2 | 10^-1^ | Circular | Raised | Entire | C.W | Glistening | P2 10^-1^ |
| 3 | 10^-1^ | Spindle | Raised | Undulate | Opaque | Dull | P3 10^-1^ |
| 1 | 10^-2^ | Circular | Raised | Entire | C.W | Smooth | P1 10^-2^ |
| 2 | 10^-2^ | Circular | Raised | Entire | Yellow | Glistening | P2 10^-2^ |
| 3 | 10^-2^ | Irregular | Raised | Undulate | Opaque | Dull | P3 10^-2^ |
| 1 | 10^-3^ | Spindle | Raised | Undulate | C.W | Smooth | P1 10^-3^ |
| 2 | 10^-3^ | Circular | Raised | Entire | C.W | Smooth | P2 10^-3^ |
| 3 | 10^-3^ | Circular | Raised | Entire | D.Y | Glistening | P3 10^-3^ |
| 1 | 10^-4^ | Spindle | Raised | Undulate | C.W | Smooth | P1 10^-4^ |
| 2 | 10^-4^ | Circular | Raised | Undulate | Opaque | Dull | P2 10^-4^ |
| 3 | 10^-4^ | Irregular | Flat | Filamentous | C.W | Smooth | P3 10^-4^ |
| 1 | 10^-5^ | Circular | Flat | Entire | C.W | Smooth | P1 10^-5^ |
| 2 | 10^-5^ | Circular | Raised | Entire | C.W | Smooth | P2 10^-5^ |
| 3 | 10^-5^ | Circular | Raised | Undulate | White | Smooth | P3 10^-5^ |
| 1 | 10^-6^ | Spindle | Flat | Undulate | White | Smooth | P1 10^-6^ |
| 2 | 10^-6^ | Irregular | Raised | Undulate | C.W | Smooth | P2 10^-6^ |
| 3 | 10^-6^ | Circular | Raised | Filamentous | Yellow | Glistening | P3 10^-6^ |
| 1 | 10^-7^ | Circular | Flat | Undulate | White | Smooth | P1 10^-7^ |
| 2 | 10^-7^ | Circular | Raised | Entire | White | Smooth | P2 10^-7^ |
| 3 | 10^-7^ | Irregular | Raised | Undulate | White | Dull | P3 10^-7^ |

C.W = Cream White; D.Y = Dark Yellow; P = Piggery/ Pig manure

**Colony morphology from the soil sub-cultures**

| **Colony no.** | **Dilution factor** | **Form** | **Elevation** | **Margin** | **Colour** | **Surface appearance** | **Organism label** | |
| --- | --- | --- | --- | --- | --- | --- | --- | --- |
| 1 | 10^-1^ | Irregular | Raised | Undulate | C.W | Glistening | S1 10^-1^ |  |
| 2 | 10^-1^ | Rhizoid | Raised | Undulate | White | Dull | S2 10^-1^ |  |
| 3 | 10^-1^ | Circular | Raised | Undulate | C.W | Glistening | S3 10^-1^ |  |
| 4 | 10^-1^ | Circular | Raised | Undulate | C.W | Glistening | S4 10^-1^ |  |
| 1 | 10^-2^ | Circular | Raised | Undulate | C.W | Glistening | S1 10^-2^ |  |
| 2 | 10^-2^ | Irregular | Flat | Undulate | C.W | Glistening | S2 10^-2^ |  |
| 3 | 10^-2^ | Circular | Raised | Entire | C.W | Rough | S3 10^-2^ |  |
| 1 | 10^-3^ | Filamentous | Raised | Filiform | White | Dull | S1 10^-3^ |  |
| 2 | 10^-3^ | Circular | Flat | Entire | Opaque | Dull | S2 10^-3^ |  |
| 3 | 10^-3^ | Irregular | Raised | Undulate | C.W | Spreading | S3 10^-3^ |  |

S = Soil

**Colony morphology from the chicken gizzard sub-cultures**

| **Colony no.** | **Dilution factor** | **Form** | **Elevation** | **Margin** | **Colour** | **Surface appearance** | **Organism label** | |
| --- | --- | --- | --- | --- | --- | --- | --- | --- |
| 1 | 10^-1^ | Filamentous | Raised | Filiform | C.W | Dry/Powdery | G1 10^-1^ |  |
| 2 | 10^-1^ | Spindle | Raised | Entire | C.W | Glistening | G2 10^-1^ |  |
| 1 | 10^-2^ | Irregular | Flat | Undulate | C.W | Smooth | G1 10^-2^ |  |
| 2 | 10^-2^ | Spindle | Raised | Undulate | C.W | Glistening | G2 10^-2^ |  |
| 1 | 10^-3^ | Irregular | Raised | Undulate | C.W | Mucoid | G1 10^-3^ |  |
| 1 | 10^-4^ | Filamentous | Raised | Filiform | C.W | Powdery | G1 10^-4^ |  |
| 2 | 10^-4^ | Spindle | Raised | Entire | C.W | Smooth | G2 10^-4^ |  |
| 1 | 10^-5^ | Circular | Raised | Undulate | C.W | Smooth | G1 10^-5^ |  |
| 1 | 10^-6^ | Spindle | Raised | Undulate | C.W | Smooth | G1 10^-6^ |  |
| 1 | 10^-7^ | Irregular | Raised | Undulate | C.W | Glistening | G1 10^-7^ |  |

G= Gizzard

**Colony morphology from the chicken small intestine sub-cultures**

| **Colony no.** | **Dilution factor** | **Form** | **Elevation** | **Margin** | **Colour** | **Surface appearance** | **Organism label** |
| --- | --- | --- | --- | --- | --- | --- | --- |
| 1 | 10^-1^ | Circular | Raised | Entire | Yellow | Mucoid | CI1 10^-1^ |
| 2 | 10^-1^ | Filamentous | Raised | Filiform | C.W | Mucoid | CI2 10^-1^ |
| 3 | 10^-1^ | Circular | Raised | Entire | C.W | Smooth | CI3 10^-1^ |
| 1 | 10^-2^ | Filamentous | Flat | Filiform | White | Dry | CI1 10^-2^ |
| 2 | 10^-2^ | Circular | Raised | Undulate | C.W | Smooth | CI2 10^-2^ |
| 1 | 10^-3^ | Filamentous | Raised | Filiform | White | Glistening | CI1 10^-3^ |
| 2 | 10^-3^ | Circular | Raised | Undulate | C.W | Smooth | CI2 10^-3^ |
| 1 | 10^-4^ | Spindle | Raised | Undulate | C.W | Smooth | CI1 10^-4^ |
| 2 | 10^-4^ | Circular | Raised | Undulate | Yellow | Glistening | CI2 10^-4^ |
| 1 | 10^-5^ | Spindle | Raised | Undulate | C.W | Smooth | CI1 10^-5^ |
| 1 | 10^-6^ | Circular | Raised | Entire | C.W | Glistening | CI1 10^-6^ |
| 1 | 10^-7^ | Spindle | Raised | Entire | White | Glistening | CI1 10^-7^ |

CI= Chicken Intestine

**Colony morphology from the raw milk sub-cultures**

| **Colony no.** | **Dilution factor** | **Form** | **Elevation** | **Margin** | **Colour** | **Surface appearance** | **Organism label** | |
| --- | --- | --- | --- | --- | --- | --- | --- | --- |
| 1 | 10^-1^ | Filamentous | Raised | Filiform | White | Smooth | M1 10^-1^ |  |
| 2 | 10^-1^ | Circular | Raised | Entire | C.W | Glistening | M2 10^-1^ |  |
| 1 | 10^-2^ | Circular | Raised | Entire | C.W | Glistening | M1 10^-2^ |  |
| 1 | 10^-3^ | Circular | Raised | Entire | C.W | Smooth | M1 10^-3^ |  |
| 1 | 10^-4^ | Irregular | Flat | Undulate | White | Smooth | M1 10^-4^ |  |
| 1 | 10^-5^ | Circular | Flat | Entire | White | Smooth | M1 10^-5^ |  |
| 1 | 10^-6^ | Irregular | Raised | Undulate | White | Glistening | M1 10^-6^ |  |
| 1 | 10^-7^ | Irregular | Raised | Undulate | C.W | Glistening | M1 10^-7^ |  |

M= Milk

**Thallus morphology from the mouldy corn sub-cultures**

| **Thallus** | **Dilution factor** | **Form** | **Elevation** | **Margin** | **Colour** | **Surface appearance** | **Organism label** |
| --- | --- | --- | --- | --- | --- | --- | --- |
| Mould | 10^-3^ | Filamentous | Raised | Filiform | Grey-green | Wrinkled | MC1 10^-3^ |
| Yeast | 10^-1^ | Circular | Raised | Entire | Yellowish | Dull | MC2 10^-1^ |

MC= Mouldy Corn

**APPENDIX II**

**Table 2.2a: Microscopic appearance of the pig manure cultures**

| **Organism** | **Gram stain reaction** | **Shape** | **Arrangement** |
| --- | --- | --- | --- |
| P1 10^-1^ | Gram-positive | Bacilli | Clusters |
| P2 10^-1^ | Gram-positive | Bacilli | Clusters |
| P3 10^-1^ | Gram-positive | Bacilli | Clusters |
| P1 10^-2^ | Gram-positive | Bacilli | Streptobacillus |
| P2 10^-2^ | Gram-positive | Bacilli | Rod |
| P3 10^-2^ | Gram-positive | Bacilli | Rod |
| P1 10^-3^ | Gram-positive | Bacilli | Rod |
| P2 10^-3^ | Gram-positive | Bacilli | Clusters |
| P3 10^-3^ | Gram-positive | Bacilli | Streptobacillus |
| P1 10^-4^ | Gram-positive | Bacilli | Clusters |
| P2 10^-4^ | Gram-positive | Bacilli | Rod |
| P3 10^-4^ | Gram-positive | Bacilli | Rod |
| P1 10^-5^ | Gram-positive | Bacilli | Clusters |
| P2 10^-5^ | Gram-positive | Bacilli | Clusters |
| P3 10^-5^ | Gram-positive | Spirilla | Clusters |
| P1 10^-6^ | Gram-positive | Bacilli | Clusters |
| P2 10^-6^ | Gram-positive | Bacilli | Clusters |
| P3 10^-6^ | Gram-positive | Spirilla | Clusters |
| P1 10^-7^ | Gram-positive | Bacilli | Clusters |
| P2 10^-7^ | Gram-positive | Bacilli | Clusters |
| P3 10^-7^ | Gram-positive | Cocci | Streptococcus |

**Table 2.2b: Microscopic appearance of the soil culture samples**

| **Organism** | **Gram stain reaction** | **Shape** | **Arrangement** |
| --- | --- | --- | --- |
| S1 10^-1^ | Gram-positive | Bacilli | Clusters |
| S2 10^-1^ | Gram-positive | Bacilli | Singularly |
| S3 10^-1^ | Gram-positive | Bacilli | Streptobacillus |
| S4 10^-1^ | Gram-positive | Bacilli | Clusters |
| S1 10^-2^ | Gram-positive | Bacilli | Singularly |
| S2 10^-2^ | Gram-positive | Bacilli | Streptobacillus |
| S3 10^-2^ | Gram-positive | Bacilli | Streptobacillus |
| S1 10^-3^ | Gram-positive | Bacilli | Streptobacillus |
| S2 10^-3^ | Gram-positive | Coccobacilli | Singularly |
| S3 10^-3^ | Gram-positive | Coccobacilli | Singularly |

**Table 2.2c: Microscopic appearance of the chicken gizzard content cultures**

| **Organism** | **Gram stain reaction** | **Shape** | **Arrangement** |
| --- | --- | --- | --- |
| G1 10^-1^ | Gram-negative | Bacilli | Singularly |
| G2 10^-1^ | Gram-negative | Bacilli | Clusters |
| G1 10^-2^ | Gram-negative | Bacilli | Streptobacillus |
| G2 10^-2^ | Gram-negative | Bacilli | Streptobacillus |
| G1 10^-3^ | Gram-negative | Bacilli | Clusters |
| G1 10^-4^ | Gram-negative | Bacilli | Streptobacillus |
| G2 10^-4^ | Gram-negative | Bacilli | Clusters |
| G1 10^-5^ | Gram-negative | Bacilli | Singularly |
| G1 10^-6^ | Gram-negative | Bacilli | Clusters |
| G1 10^-7^ | Gram-negative | Bacilli | Clusters |

**Table 2.2d: Microscope results of the chicken intestine cultures**

| **Organism** | **Gram stain reaction** | **Shape** | **Arrangement** |
| --- | --- | --- | --- |
| CI1 10^-1^ | Gram-positive | Bacilli | Singularly |
| CI2 10^-1^ | Gram-negative | Bacilli | Clusters |
| CI3 10^-1^ | Gram-negative | Bacilli | Clusters |
| CI1 10^-2^ | Gram-positive | Bacilli | Singularly |
| CI2 10^-2^ | Gram-negative | Bacilli | Streptobacillus |
| CI1 10^-3^ | Gram-negative | Bacilli | Grouped |
| CI2 10^-3^ | Gram-negative | Bacilli | Singularly |
| CI1 10^-4^ | Gram-negative | Bacilli | Singularly |
| CI2 10^-4^ | Gram-negative | Bacilli | Grouped |
| CI1 10^-5^ | Gram-negative | Cocci | Streptococcus |
| CI1 10^-6^ | Gram-negative | Bacilli | Clusters |
| CI1 10^-7^ | Gram-negative | Bacilli | Singularly |

The table presents the Gram staining results, and cell morphology of chicken intestines isolates (CI1, CI2 and CI3) at various dilution factors.

**Table 2.2e: Cellular morphology of the isolates from raw milk cultures**

| **Organism** | **Gram stain reaction** | **Shape** | **Arrangement** |
| --- | --- | --- | --- |
| M1 10^-1^ | Gram-positive | Bacilli | Streptobacillus |
| M2 10^-1^ | Gram-negative | Bacilli | Singularly |
| M1 10^-2^ | Gram-negative | Bacilli | Clusters |
| M2 10^-2^ | Gram-negative | Bacilli | Singularly |
| M1 10^-3^ | Gram-negative | Bacilli | Streptobacillus |
| M1 10^-4^ | Gram-negative | Bacilli | Streptobacillus |
| M1 10^-5^ | Gram-negative | Cocci | Clusters |
| M1 10^-6^ | Gram-negative | Bacilli | Streptobacillus |
| M1 10^-7^ | Gram-negative | Bacilli | Singularly |

The table presents the Gram staining results and cell morphology of milk isolates (M1 and M2) at various dilution factors.

**Table 2.2f: Microscope results of the mouldy maize culture**

| **Organism** | **Hyphae characteristic** | **Spore formation** | **Sporangium shape** |
| --- | --- | --- | --- |
| MC1 10^-3^ | Aseptate | Sporangiophore bear sporangia containing sporangiospores. | Oval |

**APPENDIX III:**

**Antibiotic susceptibility of ZEN-degrading isolates determined by disc diffusion assay**

| **Isolate** | **Antibiotics Disc (Mastring M14)** | | | | | | | | | |
| --- | --- | --- | --- | --- | --- | --- | --- | --- | --- | --- |
|  | **AP** | **CO** | **GM** | | **KF** | **S** | **ST** | | **T** | **TS** |
| **CC1** | NZ | NZ | 23 | | 12 | NZ | 14 | | 24 | 29 |
| **CC2** | NZ | 13 | 16 | | NZ | 11 | NZ | | 14 | 22 |
| **CI1** | NZ | 14 | 18 | | NZ | 14 | 20 | | 14 | 23 |
| **CI2** | NZ | 13 | 17 | | NZ | 12 | 19 | | 14 | 24 |
| **CI3** | NZ | NZ | 24 | | 18 | 12 | 19 | | 25 | 29 |
| **CI4** | NG | NG | NG | | NG | NG | NG | | NG | NG |
| **CI5** | NG | NG | NG | | NG | NG | NG | | NG | NG |
| **MI1** | NZ | NZ | 24 | | NZ | 21 | 26 | | 16 | 21 |
| **MI2** | NZ | 13 | 16 | | NZ | 12 | 22 | | 14 | 24 |
| **MI3** | NZ | 12 | 16 | | NZ | 12 | NZ | | 15 | 25 |
| **PI1** | NZ | 14 | 32 | | 21 | 21 | NZ | | 10 | 30 |
| **PI2** | 12 | 14 | 29 | | NZ | NZ | 28 | | 26 | 26 |
| **SO1** | NZ | 16 | 30 | | 17 | 23 | 31 | | 23 | 32 |
| **SO3** | 14 | 13 | 29 | | 14 | 21 | 30 | | 32 | 23 |
| **Isolate** | **Antibiotic Disc (Mastring M51)** | | | | | | | | | |
|  | **AP** | **AUG** | **CFX** | | **CIP** | **GM** | **NA** | | **NI** | **TM** |
| **CI4** | NG | NG | NG | | NG | NG | NG | | NG | NG |
| **PI3** | NZ | 22 | NZ | | 26 | 24 | 21 | | NZ | NZ |
| **PI4** | NZ | NZ | NZ | | 19 | 20 | 15 | | NZ | NZ |
| **Isolate** | **Single Antibiotic Disc** | | | | | | | | | |
|  | **AX** | | | **AZM** | | | | **CLX** | | |
| **CI5** | NZ | | | 33 | | | | 34 | | |

NZ= No Zone; NG= No Growth

**APPENDIX IV: CALCULATIONS**

This appendix shows calculations on the ZEN-degradation rate for the different isolates.

%ZEN = $\left\{ 1-\left[ \frac{ZEN peak area of sample}{ZEN peak area of standard} \right] \right\}x 100$

%ZEN_CI1_ = $\left\{ 1-\left[ \frac{376.7}{2709.3} \right] \right\} x 100$

= 86.09%

%ZEN_CI2_ = $\left\{ 1-\left[ \frac{427.9}{2709.3} \right] \right\} x 100$

= 84.21%

%ZEN_CI3_ = $\left\{ 1-\left[ \frac{46.5}{2709.3} \right] \right\} x 100$

= 98.28%

%ZEN_CI4_ = $\left\{ 1-\left[ \frac{246.8}{2709.3} \right] \right\} x 100$

= 90.89%

%ZEN_CI5_ = $\left\{ 1-\left[ \frac{15.1}{2709.3} \right] \right\} x 100$

= 99.44%

%ZEN_CC1_ = $\left\{ 1-\left[ \frac{82.2}{2709.3} \right] \right\} x 100$

= 96.97%

%ZEN_CC2_ = $\left\{ 1-\left[ \frac{16.2}{2709.3} \right] \right\} x 100$

= 99.40%

%ZEN_MI1_ = $\left\{ 1-\left[ \frac{17.3}{2709.3} \right] \right\} x 100$

= 99.36%

%ZEN_MI2_ = $\left\{ 1-\left[ \frac{39.6}{2709.3} \right] \right\} x 100$

= 98.54%

%ZEN_MI3_ = $\left\{ 1-\left[ \frac{26.4}{2709.3} \right] \right\} x 100$

= 99.03%

%ZEN_MC1_ = $\left\{ 1-\left[ \frac{61.8}{2709.3} \right] \right\} x 100$

= 97.72%

%ZEN_PI1_ = $\left\{ 1-\left[ \frac{103.2}{2709.3} \right] \right\} x 100$

= 96.19%

%ZEN_PI2_ = $\left\{ 1-\left[ \frac{36.0}{2709.3} \right] \right\} x 100$

= 98.67%

%ZEN_PI3_ = $\left\{ 1-\left[ \frac{18.8}{2709.3} \right] \right\} x 100$

= 99.31%

%ZEN_PI4_ = $\left\{ 1-\left[ \frac{13.1}{2709.3} \right] \right\} x 100$

= 99.52%

ZEN_SO1_ = $\left\{ 1-\left[ \frac{11.3}{2709.3} \right] \right\} x 100$

= 99.58%

ZEN_SO3_ = $\left\{ 1-\left[ \frac{83.5}{2709.3} \right] \right\} x 100$

= 96.92%
