# Supplementary material for: Effective degradation of zearalenone by multiple microbial isolates
Source: PeerJ. 2026 Apr 3;14:e20920. doi: 10.7717/peerj.20920 (PMC13052100; doi:10.7717/peerj.20920)
Supplement: Supplemental Information 3 [file peerj-14-20920-s003.docx]

>lcl|Query_95336 CC1

ACGGGGACTATACATGCAGTCGAGCGGACAGATGGGAGCTTGCTCCCTGATGTTAGCGGCGGACGGGTGAGTAACACGTGGGTAACCTGCCTGTAAGACTGGGATAACTCCGGGAAACCGGGGCTAATACCGGATGGTTGTTTGAACCGCATGGTTCAAACATAAAAGGTGGCTTCGGCTACCACTTACAGATGGACCCGCGGCGCATTAGCTAGTTGGTGAGGTAACGGCTCACCAAGGCAACGATGCGTAGCCGACCTGAGAGGGTGATCGGCCACACTGGGACTGAGACACGGCCCAGACTCCTACGGGAGGCAGCAGTAGGGAATCTTCCGCAATGGACGAAAGTCTGACGGAGCAACGCCGCGTGAGTGATGAAGGTTTTCGGATCGTAAAGCTCTGTTGTTAGGGAAGAACAAGTACCGTTCGAATAGGGCGGTACCTTGACGGTACCTAACCAGAAAGCCACGGCTAACTACGTGCCAGCAGCCGCGGTAATACGTAGGTGGCAAGCGTTGTCCGGAATTATTGGGCGTAAAGGGCTCGCAGGCGGTTTCTTAAGTCTGATGTGAAAGCCCCCGGCTCAACCGGGGAGGGTCATTGGAAACTGGGGAACTTGAGTGCAGAAGAGGAGAGTGGAATTCCACGTGTAGCGGTGAAATGCGTAGAGATGTGGAGGAACACCAGTGGCGAAGGCGACTCTCTGGTCTGTAACTGACGCTGAGGAGCGAAAGCGTGGGGAGCGAACAGGATTAGATACCCTGGTAGTCCACGCCGTAAACGATGAGTGCTAAGTGTTAGGGGGTTTCCGCCCCTTAGTGCTGCAGCTAACGCATTAAGCACTCCGCCTGGGGAGTACGGTCGCAAGACTGAAACTCAAAGGAATTGACGGGGGCCCGCACAAGCGGTGGAGCATGTGGTTTAATTCGAAGCAACGCGAAACCTTACCAGTCTTGGACATCCTCTGACAATCCTAGAGATAGGACGTCCCCTTCGGGGCATGACAGTGGTGCATGATGTCGTCAGCTCGTGTCGTGAATGTTGGGTTAGTCCGCACGACGCACCCTTGATCTAGTGCAGCATCAGTGGGCTCTAGGACTGCGGTGACAACGAGAGTGGATGACGTCAATCATCTATGCCTATGACCTGATACACCGGTCTCATGACGACATAGGCAGCGACGCCGTAAGCGATTCCAAAT

>(KX269838.1)Bacillus subtilis

ACGGGTGCTATACATGCAGTCGAGCGGACAGATGGGAGCTTGCTCCCTGATGTTAGCGGCGGACGGGTGAGTAACACGTGGGTAACCTGCCTGTAAGACTGGGATAACTCCGGGAAACCGGGGCTAATACCGGATGGTTGTTTGAACCGCATGGTTCAAACATAAAAGGTGGCTTCGGCTACCACTTACAGATGGACCCGCGGCGCATTAGCTAGTTGGTGAGGTAACGGCTCACCAAGGC-ACGATGCGTAGCCGACCTGAGAGGGTGATCGGCCACACTGGGACTGAGACACGGCCCAGACTCCTACGGGAGGCAGCAGTAGGGAATCTTCCGCAATGGACGAAAGTCTGACGGAGCAACGCCGCGTGAGTGATGAAGGTTTTCGGATCGTAAAGCTCTGTTGTTAGGGAAGAACAAGTACCGTTCGAATAGGGCGGTACCTTGACGGTACCTAACCAGAAAGCCACGGCTAACTACGTGCCAGCAGCCGCGGTAATACGTAGGTGGCAAGCGTTGTCCGGAATTATTGGGCGTAAAGGGCTCGCAGGCGGTTTCTTAAGTCTGATGTGAAAGCCCCCGGCTCAACCGGGGAGGGTCATTGGAAACTGGGGAACTTGAGTGCAGAAGAGGAGAGTGGAATTCCACGTGTAGCGGTGAAATGCGTAGAGATGTGGAGGAACACCAGTGGCGAAGGCGACTCTCTGGTCTGTAACTGACGCTGAGGAGCGAAAGCGTGGGGAGCGAACAGGATTAGATACCCTGGTAGTCCACGCCGTAAACGATGAGTGCTAAGTGTTAGGGGGTTTCCGCCCCTTAGTGCTGCAGCTAACGCATTAAGCACTCCGCCTGGGGAGTACGGTCGCAAGACTGAAACTCAAAGGAATTGACGGGGGCCCGCACAAGCGGTGGAGCATGTGGTTTAATTCGAAGCAACGCGAAACCTTACCAGTCTT-GACATCCTCTGACAATCCTAGAGATAGGACGTCCCCTTCGGGGCATGACAGTGGTGCATGGTGTCGTCAGCTCGTGTCGTGAATGT-GGGTAAGTCCGCACGACGCAACCTTGATCTAGTGCAGCATCAGTGGGCTCTAGGACTGCG-TGACAACGAGAGTGGATGACGTCAATCAT--------------------------------------------------------------------

>lcl|Query_62968 CI1

TTGGGGAGCTACACATGCAGTCGTACAGGACAAGCTTGCTGCTTGCTGACGAGTGGCGGACGGGTGAGTAATGTCTGGGAAACTGCCTGATGGAGGGGGATAACTACTGGAAACGGTAGCTAATACCGCATAACGTCGCAAGACCAAAGAGGGGGACCTTCGGGCCTCTTGCCATCGGATGTGCCCAGATGGGATTAGCTAGTAGGTGGGGTAACGGCTCACCTAGGCGACGATCCCTAGCTGGTCTGAGAGGATGACCAGCCACACTGGAACTGAGACACGGTCCAGACTCCTACGGGAGGCAGCAGTGGGGAATATTGCACAATGGGCGCAAGCCTGATGCAGCCATGCCGCGTGTATGAAGAAGGCCTTCGGGTTGTAAAGTACTTTCAGCGGGGAGGAAGGCGACAGGGTTAATAACCCTGTCGATTGACGTTACCCGCAGAAGAAGCACCGGCTAACTCCGTGCCAGCAGCCGCGGTAATACGGAGGGTGCAAGCGTTAATCGGAATTACTGGGCGTAAAGCGCACGCAGGCGGTCTGTCAAGTCGGATGTGAAATCCCCGGGCTCAACCTGGGAACTGCATTCGAAACTGGCAGGCTAGAGTCTTGTAGAGGGGGGTAGAATTCCAGGTGTAGCGGTGAAATGCGTAGAGATCTGGAGGAATACCGGTGGCGAAGGCGGCCCCCTGGACAAAGACTGACGCTCAGGTGCGAAAGCGTGGGGAGCAAACAGGATTAGATACCCTGGTAGTCCACGCCGTAAACGATGTCGACTTGGAGGTTGTGCCCTTGAGCGTGGCTTCCGGAGCTAACGCGTTAAGTCGACCGCCTGGGGAGTACGGCCGCAAGGTTAAAACTCAATGAATTGACGGGGGCCCGCACAAGCGGTGGAGCATGGTTTAATTCGATGCACGCGAGAACCTTACCTACTCTTGACATCCAAACTTACAGAGATGCTTGGTGCCTTCGGACTTGAAAGTGCTGCATGCTGTCGTCAGCTCGGTGGAATGTTGGTAAGTCGCAACGAGCACCTTATTCTTTGTGCAGCGTAGCGTACTCAGGAACTGCAGGATAACTGAGAAGTGGGATACTCAGTCATCATTGGCCTTACGATAGCTCACACGGCTCAATGCTACA

>(OP413041.1) Enterobacter hormaechei.

TTGGGGAGCTACACATGCAGTCGTACAGGAGCAGCTTGCTGCTTGCTGACGAGTGGCGGACGGGTGAGTAATGTCTGGGAAACTGCCTGATGGAGGGGGATAACTACTGGAAACGGTAGCTAATACCGCATAACGTCGCAAGACCAAAGAGGGGGACCTTCGGGCCTCTTGCCATCGGATGTGCCCAGATGGGATTAGCTAGTAGGTGGGGTAACGGCTCACCTAGGCGACGATCCCTAGCTGGTCTGAGAGGATGACCAGCCACACTGGAACTGAGACACGGTCCAGACTCCTACGGGAGGCAGCAGTGGGGAATATTGCACAATGGGCGCAAGCCTGATGCAGCCATGCCGCGTGTATGAAGAAGGCCTTCGGGTTGTAAAGTACTTTCAGCGGGGAGGAAGGCGACAGGGTTAATAACCCTGTCGATTGACGTTACCCGCAGAAGAAGCACCGGCTAACTCCGTGCCAGCAGCCGCGGTAATACGGAGGGTGCAAGCGTTAATCGGAATTACTGGGCGTAAAGCGCACGCAGGCGGTCTGTCAAGTCGGATGTGAAATCCCCGGGCTCAACCTGGGAACTGCATTCGAAACTGGCAGGCTAGAGTCTTGTAGAGGGGGGTAGAATTCCAGGTGTAGCGGTGAAATGCGTAGAGATCTGGAGGAATACCGGTGGCGAAGGCGGCCCCCTGGACAAAGACTGACGCTCAGGTGCGAAAGCGTGGGGAGCAAACAGGATTAGATACCCTGGTAGTCCACGCCGTAAACGATGTCGACTTGGAGGTTGTGCCCTTGAGCGTGGCTTCCGGAGCTAACGCGTTAAGTCGACCGCCTGGGGAGTACGGCCGCAAGGTTAAAACTCAATGAATTGACGGGGGCCCGCACAAGCGGTGGAGCATGGTTTAATTCGATGCACGCGAGAACCTTACCTACTCTTGACATCCAAACTTACAGAGATGCTTGGTGCCTTCGGACTTGAAAGTGCTGCATGCTGTCGTCAGCTCGGTGGAATGTTGGTAAGTCGCAACGAGCACCTTATCCTTTGTGCAGCGTAGCGGACTCAGGAACTGCAGGATAACTGAGAAGTGGGATACTCAGTCATCATGGCCCTTACGATAGCTCACACGGCTCAATG-----

>lcl|Query_128088 CC2

CGGGGGGGGCTACACATGCAGTCGTACAAAACGCAACAGCTTGCTGCTTGCTGACGAGTGGCGGACGGGTGAGTAATGTCTGGGAAACTGCCTGATGGAGGGGGATAACTACTGGAAACGGTAGCTAATACCGCATAACGTCGCAAGACCAAAGAGGGGGACCTTCGGGCCTCTTGCCATCGGATGTGCCCAGATGGGATTAGCTAGTAGGTGGGGTAACGGCTCACCTAGGCGACGATCCCTAGCTGGTCTGAGAGGATGACCAGCCACACTGGAACTGAGACACGGTCCAGACTCCTACGGGAGGCAGCAGTGGGGAATATTGCACAATGGGCGCAAGCCTGATGCAGCCATGCCGCGTGTATGAAGAAGGCCTTCGGGTTGTAAAGTACTTTCAGCGGGGAGGAAGGCGACAGGGTTAATAACCCTGTCGATTGACGTTACCCGCAGAAGAAGCACCGGCTAACTCCGTGCCAGCAGCCGCGGTAATACGGAGGGTGCAAGCGTTAATCGGAATTACTGGGCGTAAAGCGCACGCAGGCGGTCTGTCAAGTCGGATGTGAAATCCCCGGGCTCAACCTGGGAACTGCATTCGAAACTGGCAGGCTAGAGTCTTGTAGAGGGGGGTAGAATTCCAGGTGTAGCGGTGAAATGCGTAGAGATCTGGAGGAATACCGGTGGCGAAGGCGGCCCCCTGGACAAAGACTGACGCTCAGGTGCGAAAGCGTGGGGAGCAAACAGGATTAGATACCCTGGTAGTCCACGCCGTAACGATGTCGACTTGGAGGTTGTGCCCTTGAGGCGTGGCTTCCGGAGCTAACGCGTTAAGTCGACCGCCTGGGGAGTACGGCCGCAAGGTTAAAACTCAAATGAATTGACGGGGGCCCGCACAAGCGGTGGAGCATGTGGTTTAATTCGATGCAACGCGAGAACCTTACCTACTCTTGACATCCAGAGACTTACCAGAATGTTGGGCCTTCGGGACTCTGAGACAGTGCTGCATGGCTGTCGTCAGCTCGTGTTGTGAATGTTGGTTAGTCCGCAACGACGCAACCTTATCTTTGTTGCAGGTGGCGGACTCAAGAACGCAGATAACTGAGAGTGGGATGACGTCAGTCTATGGCTACGATAGCTAC

>(OQ970511.1) Enterobacter sp..

CGGGGGCGGCTACACATGCAGTCGAACGTAACGGAACAGCTTGCTGCTTGCTGACGAGTGGCGGACGGGTGAGTAATGTCTGGGAAACTGCCTGATGGAGGGGGATAACTACTGGAAACGGTAGCTAATACCGCATAACGTCGCAAGACCAAAGAGGGGGACCTTCGGGCCTCTTGCCATCGGATGTGCCCAGATGGGATTAGCTAGTAGGTGGGGTAACGGCTCACCTAGGCGACGATCCCTAGCTGGTCTGAGAGGATGACCAGCCACACTGGAACTGAGACACGGTCCAGACTCCTACGGGAGGCAGCAGTGGGGAATATTGCACAATGGGCGCAAGCCTGATGCAGCCATGCCGCGTGTATGAAGAAGGCCTTCGGGTTGTAAAGTACTTTCAGCGGGGAGGAAGGCGACAGGGTTAATAACCCTGTCGATTGACGTTACCCGCAGAAGAAGCACCGGCTAACTCCGTGCCAGCAGCCGCGGTAATACGGAGGGTGCAAGCGTTAATCGGAATTACTGGGCGTAAAGCGCACGCAGGCGGTCTGTCAAGTCGGATGTGAAATCCCCGGGCTCAACCTGGGAACTGCATTCGAAACTGGCAGGCTAGAGTCTTGTAGAGGGGGGTAGAATTCCAGGTGTAGCGGTGAAATGCGTAGAGATCTGGAGGAATACCGGTGGCGAAGGCGGCCCCCTGGACAAAGACTGACGCTCAGGTGCGAAAGCGTGGGGAGCAAACAGGATTAGATACCCTGGTAGTCCACGCCGTAACGATGTCGACTTGGAGGTTGTGCCCTTGAGGCGTGGCTTCCGGAGCTAACGCGTTAAGTCGACCGCCTGGGGAGTACGGCCGCAAGGTTAAAACTCAAATGAATTGACGGGGGCCCGCACAAGCGGTGGAGCATGTGGTTTAATTCGATGCAACGCGAGAACCTTACCTACTCTTGACATCCAGAGACTTTCCAGAATGTTGGGCCTTCGGGACTCTGAGACAGTGCTGCATGGCTGTCGTCAGCTCGTGTTGTGAATGTTGGTTAGTCCGCAACGACGCAACCTTATCTTTGTTGC--------------------------------------------------------------------

>lcl|Query_487844 CI5

TGGGGGGCTAATAATGCAGTCGAGCGAACTGATTAGAAGCTTGCTTCTATGACGTTAGCGGCGGACGGGTGAGTAACACGTGGGCAACCTGCCTGTAAGACTGGGATAACTTCGGGAAACCGAAGCTAATACCGGATAGGATCTTCTCCTTCATGGGAGATGATTGAAAGATGGTTTCGGCTATCACTTACAGATGGGCCCGCGGTGCATTAGCTAGTTGGTGAGGTAACGGCTCACCAAGGCAACGATGCATAGCCGACCTGAGAGGGTGATCGGCCACACTGGGACTGAGACACGGCCCAGACTCCTACGGGAGGCAGCAGTAGGGAATCTTCCGCAATGGACGAAAGTCTGACGGAGCAACGCCGCGTGAGTGATGAAGGCTTTCGGGTCGTAAAACTCTGTTGTTAGGGAAGAACAAGTACGAGAGTAACTGCTCGTACCTTGACGGTACCTAACCAGAAAGCCACGGCTAACTACGTGCCAGCAGCCGCGGTAATACGTAGGTGGCAAGCGTTATCCGGAATTATTGGGCGTAAAGCGCGCGCAGGCGGTTTCTTAAGTCTGATGTGAAAGCCCACGGCTCAACCGTGGAGGGTCATTGGAAACTGGGGAACTTGAGTGCAGAAGAGAAAAGCGGAATTCCACGTGTAGCGGTGAAATGCGTAGAGATGTGGAGGAACACCAGTGGCGAAGGCGGCTTTTTGGTCTGTAACTGACGCTGAGGCGCGAAAGCGTGGGGAGCAAACAGGATTAGATACCCTGGTAGTCCACGCCGTAAACGATGAGTGCTAAGTGTTAGAGGGTTTCCGCCCTTTAGTGCTGCAGCTAACGCATTAAGCACTCCGCCTGGGGAGTACGGTCGCAAGACTGAAACTCAAAGGAATTGACGGGGGCCCGCACAAGCGGTGGAGCATGTGGTTTAATTCGAAGCAACGCGAAGAACCTTACCAGGGTCTTGACATCCTCTGAACTCTAGAATAAGCGTTCCCCTTCGGGGAAAGTGAACAGGTGGTGCATGATGTCGTCAGCTCGTGTCGTGAATGTGGGTAGTCCGCACGCCACCCTGATCTAGTGCAGCATTTAGTGGCACTCTAGTGACTGCGTGACAACGAGAAGGTGGGGACACGTCAATCATCATGCCTTGTACCTGCTACGTCTCAATTGATGTCAGCGCAGAACCGCGAAGTCCAGCAT

>(OR234783.1) Bacillus sp. (in: firmicutes) .

-GGGGGGCCTATACTGCAGTCGAGCGAACTGATTAGAAGCTTGCTTCTATGACGTTAGCGGCGGACGGGTGAGTAACACGTGGGCAACCTGCCTGTAAGACTGGGATAACTTCGGGAAACCGAAGCTAATACCGGATAGGATCTTCTCCTTCATGGGAGATGATTGAAAGATGGTTTCGGCTATCACTTACAGATGGGCCCGCGGTGCATTAGCTAGTTGGTGAGGTAACGGCTCACCAAGGCAACGATGCATAGCCGACCTGAGAGGGTGATCGGCCACACTGGGACTGAGACACGGCCCAGACTCCTACGGGAGGCAGCAGTAGGGAATCTTCCGCAATGGACGAAAGTCTGACGGAGCAACGCCGCGTGAGTGATGAAGGCTTTCGGGTCGTAAAACTCTGTTGTTAGGGAAGAACAAGTACGAGAGTAACTGCTCGTACCTTGACGGTACCTAACCAGAAAGCCACGGCTAACTACGTGCCAGCAGCCGCGGTAATACGTAGGTGGCAAGCGTTATCCGGAATTATTGGGCGTAAAGCGCGCGCAGGCGGTTTCTTAAGTCTGATGTGAAAGCCCACGGCTCAACCGTGGAGGGTCATTGGAAACTGGGGAACTTGAGTGCAGAAGAGAAAAGCGGAATTCCACGTGTAGCGGTGAAATGCGTAGAGATGTGGAGGAACACCAGTGGCGAAGGCGGCTTTTTGGTCTGTAACTGACGCTGAGGCGCGAAAGCGTGGGGAGCAAACAGGATTAGATACCCTGGTAGTCCACGCCGTAAACGATGAGTGCTAAGTGTTAGAGGGTTTCCGCCCTTTAGTGCTGCAGCTAACGCATTAAGCACTCCGCCTGGGGAGTACGGTCGCAAGACTGAAACTCAAAGGAATTGACGGGGGCCCGCACAAGCGGTGGAGCATGTGGTTTAATTCGAAGCAACGCGAAGAACCTTACCAGGTTCTTGACATCCTCTGAACTCTAGAATAAGCGTTTCCCTTCGGGGAAAGTG-ACAGGTGGTGCATGTTGTCGTCAGCTCGTGTCGTGAATGTTGGTAGTCCGCACGCCACCCTGATCTAGTGCAGCA-TTAGTGGCACTCTAGTGACTGCGTGACA-CGAGA-GGTGGGAATACGTCAATCATCATGCCTTAGACCTGCTACGTCTCAATTGATGT--------------------------

>lcl|Query_33486 CI3

ATGGGTGACTTGCTCCCTGATGTTACGGGGGACGGGGATAACACGTGGGTAACCTGCTGTAAGATGGGATAACTCCGGGAAACCGGGGCTATACCGGATGGTTGTTGAACCGCATGGTTCAAACATAAAAGGTGGCTTCGGCTACCACTTACAGATGGACCCGCGGCGCATTAGCTAGTTGGTGAGGTAAGGTCACAAGACGATGCGTACGACCTGAGAGGGGATCGGCCCACTGGGACTGAACACGGCCCAGACTCCTACGGGAGGCAGCAGTAGGGAATCTTCCGCAATGGACGAAAGTCTGACGGAGCAACGCCGCGTGAGTGATGAAGGTTTTCGGATCGTAAAGCTCTGTTGTTAGGGAAGAACAAGTACCGTTCGAATAGGGCGGTACCTTGACGGTACCTAACCAGAAAGCCACGGCTAACTACGTGCCAGCAGCCGCGGTAATACGTAGGGAAGGTTGTCCGGAATTATTGGGCGTAAAGGGCTCGCAGGCGGTTTCTTAAGTCTGATGTGAAAGCCCCCGGCTCAACCGGGGAGGGTCATTGGAAACTGGGGAACTTGAGTGCAGAAGAGGAGAGTGGAATTCCACGTGTAGCGGTGAAATGCGGTAGAGATGTGGAGGAACACCAGTGGGCGAAGGCGACTCTCTGGTCTGTAACTGACGCTGAGGAGCGAAAGCGTGGGGAGCGAACAGGATTAGATACCCTGGTAGTCCACGCCGTAAACGATGAGTGCTAAGTGTTAGGGGGTTTCCGCCCCTTAGTGCTGCAGCTAAGCATTAAGCACTCCGCCTGGGGAGTACGGTGCAAGACTGAAACTCAAAGAATTGACGGGGGCCCGCACAGCGGGGAGCATGTGGTTAATTCGAAGCAACGAAACCTTACCAGGTCTTGAATCCGACATCCTAAAATAGAACGTCCCTTCGGGGGAAAGAAGGGTGCATGGGTGTCGCAGCTTCGGTCTAATGTGGATAAGTCCCACGACGCAACCATGATCTAGTGCACTCATGGGCACTAAGGGAGCCGGGGACAACCGAGAGAGGTGGGGAAAGCTCAATCTCATGGCCCTTTAGACTGGCTAGCCGGCTATCATGAC

>(MK367792.1) Bacillus subtilis

ATGGGAG-CTTGCTCCCTGATGTTACGGCGGACGGGGATAACACGTGGGTAACCTGCTGTAAGATGGGATAACTCCGGGAAACCGGGGCTATACCGGATGGTTGTTGAACCGCATGGTTCAAACATAAAAGGTGGCTTCGGCTACCACTTACAGATGGACCCGCGGCGCATTAGCTAGTTGGTGAGGTAAGGTCACAAGACGATGCGTACGACCTGAGAGGGGATCGGCCCACTGGGACTGAACACGGCCCAGACTCCTACGGGAGGCAGCAGTAGGGAATCTTCCGCAATGGACGAAAGTCTGACGGAGCAACGCCGCGTGAGTGATGAAGGTTTTCGGATCGTAAAGCTCTGTTGTTAGGGAAGAACAAGTACCGTTCGAATAGGGCGGTACCTTGACGGTACCTAACCAGAAAGCCACGGCTAACTACGTGCCAGCAGCCGCGGTAATACGTAGGGAAGGTTGTCCGGAATTATTGGGCGTAAAGGGCTCGCAGGCGGTTTCTTAAGTCTGATGTGAAAGCCCCCGGCTCAACCGGGGAGGGTCATTGGAAACTGGGGAACTTGAGTGCAGAAGAGGAGAGTGGAATTCCACGTGTAGCGGTGAAATGC-GTAGAGATGTGGAGGAACACCAGT-GGCGAAGGCGACTCTCTGGTCTGTAACTGACGCTGAGGAGCGAAAGCGTGGGGAGCGAACAGGATTAGATACCCTGGTAGTCCACGCCGTAAACGATGAGTGCTAAGTGTTAGGGGGTTTCCGCCCCTTAGTGCTGCAGCTAAGCATTAAGCACTCCGCCTGGGGAGTACGGTGCAAGACTGAAACTCAAAGAATTGACGGGGGCCCGCACAGCGGGGAGCATGTGGTTAATTCGAAGCAACGAAACCTTACCAGGTCTTGAATCCGACATCCTAGAATAGGACGTCCCTTCGGGGGCAAGAAGGGTGCATGATTGTCGCAGCT-CGGTCTAATGTGGGTAAGTCCCACGACGCAACCCTGATCTAGTGCACTCATGGGCACTAAGGGA-CTGGGGACAACGGAGA-AGGTGGGGATACGTCAATCTCATGCCCCTT--------------------------

>lcl|Query_76954 CI4

GGGGGGGAGCTAACACATGCAGTCGAAAAACGCAACAGCTTGCTGCTTGCTGACGAGTGGCGGACGGGTGAGTAATGTCTGGGAAACTGCCTGATGGAGGGGGATAACTACTGGAAACGGTAGCTAATACCGCATAACGTCGCAAGACCAAAGAGGGGGACCTTCGGGCCTCTTGCCATCGGATGTGCCCAGATGGGATTAGCTAGTAGGTGGGGTAACGGCTCACCTAGGCGACGATCCCTAGCTGGTCTGAGAGGATGACCAGCCACACTGGAACTGAGACACGGTCCAGACTCCTACGGGAGGCAGCAGTGGGGAATATTGCACAATGGGCGCAAGCCTGATGCAGCCATGCCGCGTGTATGAAGAAGGCCTTCGGGTTGTAAAGTACTTTCAGCGGGGAGGAAGGCGACAGGGTTAATAACCCTGTCGATTGACGTTACCCGCAGAAGAAGCACCGGCTAACTCCGTGCCAGCAGCCGCGGTAATACGGAGGGTGCAAGCGTTAATCGGAATTACTGGGCGTAAAGCGCACGCAGGCGGTCTGTCAAGTCGGATGTGAAATCCCCGGGCTCAACCTGGGAACTGCATTCGAAACTGGCAGGCTAGAGTCTTGTAGAGGGGGGTAGAATTCCAGGTGTAGCGGTGAAATGCGTAGAGATCTGGAGGAATACCGGTGGCGAAGGCGGCCCCCTGGACAAAGACTGACGCTCAGGTGCGAAAGCGTGGGGAGCAAACAGGATTAGATACCCTGGTAGTCCACGCCGTAAACGATGTCGACTTGGAGGTTGTGCCCTTGAGGCGTGGCTTCCGGAGCTAACGCGTTAAGTCGACCGCCTGGGGAGTACGGCCGCAAGGTTAAAACTCAAATGAATTGACGGGGGCCCGCACAAGCGGGAGCATGTGGTTTAATTCGATGCAACGCGAAACCTTACCTACTCTTGACATCCAGAGAACTTACAGAGATGCATTGGTGCCTTCGGGACTCTGAACAGGTGCTGCATGGCTGTCGTCAGCTCGTGTGTGAATGTGGTAGTCCGCACGAGCGCACCCTTATTCTTTGTGCAGGTTGGCGGAACTCAAGGAACTGCAGTGATAACTGAGAGGGGGGATGATCAAGTCTCATGCTACATAGGCTACAC

>(OP413043.1) Enterobacter hormaechei

GGGCGGCAGCT-ACACATGCAGTCGAATAACGGAACAGCTTGCTGCTTGCTGACGAGTGGCGGACGGGTGAGTAATGTCTGGGAAACTGCCTGATGGAGGGGGATAACTACTGGAAACGGTAGCTAATACCGCATAACGTCGCAAGACCAAAGAGGGGGACCTTCGGGCCTCTTGCCATCGGATGTGCCCAGATGGGATTAGCTAGTAGGTGGGGTAACGGCTCACCTAGGCGACGATCCCTAGCTGGTCTGAGAGGATGACCAGCCACACTGGAACTGAGACACGGTCCAGACTCCTACGGGAGGCAGCAGTGGGGAATATTGCACAATGGGCGCAAGCCTGATGCAGCCATGCCGCGTGTATGAAGAAGGCCTTCGGGTTGTAAAGTACTTTCAGCGGGGAGGAAGGCGACAGGGTTAATAACCCTGTCGATTGACGTTACCCGCAGAAGAAGCACCGGCTAACTCCGTGCCAGCAGCCGCGGTAATACGGAGGGTGCAAGCGTTAATCGGAATTACTGGGCGTAAAGCGCACGCAGGCGGTCTGTCAAGTCGGATGTGAAATCCCCGGGCTCAACCTGGGAACTGCATTCGAAACTGGCAGGCTAGAGTCTTGTAGAGGGGGGTAGAATTCCAGGTGTAGCGGTGAAATGCGTAGAGATCTGGAGGAATACCGGTGGCGAAGGCGGCCCCCTGGACAAAGACTGACGCTCAGGTGCGAAAGCGTGGGGAGCAAACAGGATTAGATACCCTGGTAGTCCACGCCGTAAACGATGTCGACTTGGAGGTTGTGCCCTTGAGGCGTGGCTTCCGGAGCTAACGCGTTAAGTCGACCGCCTGGGGAGTACGGCCGCAAGGTTAAAACTCAAATGAATTGACGGGGGCCCGCACAAGCGGGAGCATGTGGTTTAATTCGATGCAACGCGAAACCTTACCTACTCTTGACATCCAGAGAACTTACAGAGATGCTTTGGTGCCTTCGGGACTCTGAACAGGTGCTGCATGGCTGTCGTCAGCTCGTGTGTGAATGTGGTAGTCCGCACGAGCGCACCCTTATCCTTTGTGCAGGTTGGCGGAACTCAAGGAACTGCAGTGATAACTGAGAGGGGGGATGATCAAGTCTCATG---------------

>lcl|Query_42688 MI2

GCGGCAGGCTACACATGCAGTCGTACAAGGACGAAGCTTGCTGCTTGCTGACGAGTGGCGGCGGGTGAGTAATGTCTGGGAAACTGCCTGATGGAGGGGGATAACTACTGGAAACGGTAGCTAATACCGCATAACGTCGCAAGACCAAAGAGGGGGACCTTCGGGCCTCTTGCCATCGGATGTGCCCAGATGGGATTAGCTAGTAGGTGGGGTAACGGCTCACCTAGGCGACGATCCCTAGCTGGTCTGAGAGGATGACCAGCCACACTGGAACTGAGACACGGTCCAGACTCCTACGGGAGGCAGCAGTGGGGAATATTGCACAATGGGCGCAAGCCTGATGCAGCCATGCCGCGTGTATGAAGAAGGCCTTCGGGTTGTAAAGTACTTTCAGCGGGGAGGAAGGCGACAGGGTTAATAACCCTGTCGATTGACGTTACCCGCAGAAGAAGCACCGGCTAACTCCGTGCCAGCAGCCGCGGTAATACGGAGGGTGCAAGCGTTAATCGGAATTACTGGGCGTAAAGCGCACGCAGGCGGTCTGTCAAGTCGGATGTGAAATCCCCGGGCTCAACCTGGGAACTGCATTCGAAACTGGCAGGCTAGAGTCTTGTAGAGGGGGGTAGAATTCCAGGTGTAGCGGTGAAATGCGTAGAGATCTGGAGGAATACCGGTGGCGAAGGCGGCCCCCTGGACAAAGACTGACGCTCAGGTGCGAAAGCGTGGGGAGCAAACAGGATTAGATACCCTGGTAGTCCACGCCGTAAACGATGTCGACTTGGAGGTTGTGCCCTTGAGCGTGGCTTCCGGAGCTAACGCGTTAAGTCGACCGCCTGGGGAGTACGGCCGCAAGGTTAAACTCAATGAATTGACGGGGGCCCGCACAGCGGTGGAGCATGTGGTTTAATTCGATGCAACGCGAAGAACCTTACCTACTCTTGACATCCGAGAACTTACCAGAGATGCATTGGTGCCTTCGGGAACTCTGAGAAGTGCTGCATGGCTGTCGTCAGCTCGTGTTGTGAAATGTGGGGTAGTTCCGCAACACGCACCCTTATCTTTGTTGCCCGGTTCAGCGGGACTCAAAGGAACTGCAGATACTGAGGAGGGGATGACGTCAGTCATCATGCCTTACGTAGGCTACCCGTGCTCATGCATCAAGAAAGCACCTCCGGAGCACGGACCTCATAGGTGCG

>(OQ970492.1) Enterobacter sp.

GCGGCAGGCTACACATGCAGTCGAACAAGGAAGCAGCTTGCTGCTTGCTGACGAGTGGCGGCGGGTGAGTAATGTCTGGGAAACTGCCTGATGGAGGGGGATAACTACTGGAAACGGTAGCTAATACCGCATAACGTCGCAAGACCAAAGAGGGGGACCTTCGGGCCTCTTGCCATCGGATGTGCCCAGATGGGATTAGCTAGTAGGTGGGGTAACGGCTCACCTAGGCGACGATCCCTAGCTGGTCTGAGAGGATGACCAGCCACACTGGAACTGAGACACGGTCCAGACTCCTACGGGAGGCAGCAGTGGGGAATATTGCACAATGGGCGCAAGCCTGATGCAGCCATGCCGCGTGTATGAAGAAGGCCTTCGGGTTGTAAAGTACTTTCAGCGGGGAGGAAGGCGACAGGGTTAATAACCCTGTCGATTGACGTTACCCGCAGAAGAAGCACCGGCTAACTCCGTGCCAGCAGCCGCGGTAATACGGAGGGTGCAAGCGTTAATCGGAATTACTGGGCGTAAAGCGCACGCAGGCGGTCTGTCAAGTCGGATGTGAAATCCCCGGGCTCAACCTGGGAACTGCATTCGAAACTGGCAGGCTAGAGTCTTGTAGAGGGGGGTAGAATTCCAGGTGTAGCGGTGAAATGCGTAGAGATCTGGAGGAATACCGGTGGCGAAGGCGGCCCCCTGGACAAAGACTGACGCTCAGGTGCGAAAGCGTGGGGAGCAAACAGGATTAGATACCCTGGTAGTCCACGCCGTAAACGATGTCGACTTGGAGGTTGTGCCCTTGAGCGTGGCTTCCGGAGCTAACGCGTTAAGTCGACCGCCTGGGGAGTACGGCCGCAAGGTTAAACTCAATGAATTGACGGGGGCCCGCACAGCGGTGGAGCATGTGGTTTAATTCGATGCAACGCGAAGAACCTTACCTACTCTTGACATCCGAGAACTTTCCAGAGATGGATTGGTGCCTTCGGGAACTCTGAGAAGTGCTGCATGGCTGTCGTCAGCTCGTGTTGTGAAATGTTGGGTAGTCCCGCAACACGCACCCTTATCTTTGTTGCCCGGTTAGGCGGGACTCAAAGGAACTGCAGATACTGAGGAGGGGATGACGTCAGTCATCATGCCTTACGTAGGCTACCCGTGCTCATGCATCAAGAAAGCACCTCCGGAGCACGGACCTCATAAGTGCG

>(CP126864.1) Enterobacter hormaechei

GCGGCAGGCTACACATGCAGTCGAACAAGGAAGCAGCTTGCTGCTTGCTGACGAGTGGCGGCGGGTGAGTAATGTCTGGGAAACTGCCTGATGGAGGGGGATAACTACTGGAAACGGTAGCTAATACCGCATAACGTCGCAAGACCAAAGAGGGGGACCTTCGGGCCTCTTGCCATCGGATGTGCCCAGATGGGATTAGCTAGTAGGTGGGGTAACGGCTCACCTAGGCGACGATCCCTAGCTGGTCTGAGAGGATGACCAGCCACACTGGAACTGAGACACGGTCCAGACTCCTACGGGAGGCAGCAGTGGGGAATATTGCACAATGGGCGCAAGCCTGATGCAGCCATGCCGCGTGTATGAAGAAGGCCTTCGGGTTGTAAAGTACTTTCAGCGGGGAGGAAGGCGACAGGGTTAATAACCCTGTCGATTGACGTTACCCGCAGAAGAAGCACCGGCTAACTCCGTGCCAGCAGCCGCGGTAATACGGAGGGTGCAAGCGTTAATCGGAATTACTGGGCGTAAAGCGCACGCAGGCGGTCTGTCAAGTCGGATGTGAAATCCCCGGGCTCAACCTGGGAACTGCATTCGAAACTGGCAGGCTAGAGTCTTGTAGAGGGGGGTAGAATTCCAGGTGTAGCGGTGAAATGCGTAGAGATCTGGAGGAATACCGGTGGCGAAGGCGGCCCCCTGGACAAAGACTGACGCTCAGGTGCGAAAGCGTGGGGAGCAAACAGGATTAGATACCCTGGTAGTCCACGCCGTAAACGATGTCGACTTGGAGGTTGTGCCCTTGAGCGTGGCTTCCGGAGCTAACGCGTTAAGTCGACCGCCTGGGGAGTACGGCCGCAAGGTTAAACTCAATGAATTGACGGGGGCCCGCACAGCGGTGGAGCATGTGGTTTAATTCGATGCAACGCGAAGAACCTTACCTACTCTTGACATCCGAGAACTTTCCAGAGATGGATTGGTGCCTTCGGGAACTCTGAGAAGTGCTGCATGGCTGTCGTCAGCTCGTGTTGTGAAATGTTGGGTAGTCCCGCAACACGCACCCTTATCTTTGTTGCCCGGTTAGGCGGGACTCAAAGGAACTGCAGATACTGAGGAGGGGATGACGTCAGTCATCATGCCTTACGTAGGCTACCCGTGCTCATGCATCAAGAAAGCACCTCCGGAGCACGGACCTCATAAGTGCG

>lcl|Query_18060 SO2

GCAGAGGGAGCTGCTCCCTGATGTTAGCGGCGGCGGTGAGTAACACGTGGGTAACCTGCCTGTAAGACTGGGATAACTCCGGGAAACCGGGGCTAATACCGGATGGTTGTTGAACCGCATGGTTCAGACATAAAAGGTGGCTTCGGCTACCACTTACAGATGGACCCGCGGCGCATTAGCTAGTTGGTGAGGTAACGTCCAAGGCACGATGCGTGCGACCTGAGAGGGTGATCGGCCACACTGGGACTGAAACGGCCCAGACTCCTACGGGAGGCAGCAGTAGGGAATCTTCCGCAATGGACGAAAGTCTGACGGAGCAACGCCGCGTGAGTGATGAAGGTTTTCGGATCGAAAGCTCTGTTGTTAGGGAAGAACAAGTGCCGTTCAAATAGGGCGGCACCTTGACGGTACCTAACCAGAAAGCCACGGCTAACTACGTGCCAGCAGCCGCGGTAATACGTAGGTGGCAAGCGTTGTCCGGAATTATTGGGCGTAAAGGGCTCGCAGGCGGTTTCTTAAGTCTGATGTGAAAGCCCCCGGCTCAACCGGGGAGGGTCATTGGAAACTGGGGAACTTGAGTGCAGAAGAGGAGAGTGGAATTCCACGTGTAGCGGTGAAATGCGTAGAGATGTGGAGGAACACCAGTGGCGAAGGCGACTCTCTGGTCTGTAACTGACGCTGAGGAGCGAAAGCGTGGGGAGCGAACAGGATTAGATACCCTGGTAGTCCACGCCGTAAACGATGAGTGCTAAGTGTTAGGGGGTTTCCGCCCCTTAGTGCTGCAGCTAACGCATTAAGCACTCCGCCTGGGGAGTACGGTCGAGACTGAACTCAAGGATTGACGGGGGCCGCACAAGCCGGGAGCATGTGGTTTATTCGAGGCACGCGGAGAACCTTACCAGGCTTGACATCCTCTGACAATCCTAAGAAGAGTCCCTTCCGGGGCAGAGGAGGTGGTGGCATGGGTGTCGTCAGCTCGCTCCTGAGAATGTTGGGGATAAGTCCGCAACGACGCAACCCTTGGACTAGTGCAGCATCGTGGCACTCTAAGGGACTGCCGGTGCACCGGAGAGGGGGGAAGTCAATAGCCGTATGAACCTGCTACGGCTCATGGACGACAAG

>(KC492052.1) Bacillus amyloliquefaciens

-CAGAGGGAGCTGCTCCCTGATGTTAGCGGCGGCGGTGAGTAACACGTGGGTAACCTGCCTGTAAGACTGGGATAACTCCGGGAAACCGGGGCTAATACCGGATGGTTGTTGAACCGCATGGTTCAGACATAAAAGGTGGCTTCGGCTACCACTTACAGATGGACCCGCGGCGCATTAGCTAGTTGGTGAGGTAACGTCCAAGGCACGATGCGTGCGACCTGAGAGGGTGATCGGCCACACTGGGACTGAAACGGCCCAGACTCCTACGGGAGGCAGCAGTAGGGAATCTTCCGCAATGGACGAAAGTCTGACGGAGCAACGCCGCGTGAGTGATGAAGGTTTTCGGATCGAAAGCTCTGTTGTTAGGGAAGAACAAGTGCCGTTCAAATAGGGCGGCACCTTGACGGTACCTAACCAGAAAGCCACGGCTAACTACGTGCCAGCAGCCGCGGTAATACGTAGGTGGCAAGCGTTGTCCGGAATTATTGGGCGTAAAGGGCTCGCAGGCGGTTTCTTAAGTCTGATGTGAAAGCCCCCGGCTCAACCGGGGAGGGTCATTGGAAACTGGGGAACTTGAGTGCAGAAGAGGAGAGTGGAATTCCACGTGTAGCGGTGAAATGCGTAGAGATGTGGAGGAACACCAGTGGCGAAGGCGACTCTCTGGTCTGTAACTGACGCTGAGGAGCGAAAGCGTGGGGAGCGAACAGGATTAGATACCCTGGTAGTCCACGCCGTAAACGATGAGTGCTAAGTGTTAGGGGGTTTCCGCCCCTTAGTGCTGCAGCTAACGCATTAAGCACTCCGCCTGGGGAGTACGGTCGAGACTGAACTCAAGGATTGACGGGGGCCGCACAAGCGGGGAGCATGTGGTTTATTCGAAGCACGCGAAGAACCTTACCAGGCTTGACATCCTCTGACAATCCTAAGAAGAGTCCCTTCGGGGGCAGAGGAGGTGGT-GCATGGTTGTCGTCAGCTCGTTCGTGAGA-TGTTGGGT-TAAGTCCGCAACGACGCAACCCTTGATCTAGTGCAGCATCGTGGCACTCTA-GTGACTGC-GGTGCACCGGAGAGGTGGGA-------------------------------------------

>lcl|Query_31336 PI3

GGGTAACCGTGCTACCTGACCTAGCGGCGGACGGGTGAGTAATGCTTAGGAATCTGCCTATTAGTGGGGGACAACTTCCGAAAGGAATGCTAATACCGCATACGCCTACGGGGGAAAGCAGGGGATCTTCGGACCTTGCGCTAATAGATGAGCCTAAGTCGGATTAGCTAGTTGGTGGGGTAAAGGCCTACCAAGGCGACGATCTGTAGCGGGTCTGAGAGGATGATCCGCCACACTGGGACTGAGACACGGCCCAGACTCCTACGGGAGGCAGCAGTGGGGAATATTGGACAATGGGCGCAAGCCTGATCCAGCCATGCCGCGTGTGTGAAGAAGGCCTTTTGGTTGTAAAGCACTTTAAGCGAGGAGGAGGCTCCTTTAGTTAATACCTAAAGGAGTGGACGTTACTCGCAGAATAAGCACCGGCTAACTCTGTGCCAGCAGCCGCGGTAATACAGAGGGTGCGAGCGTTAATCGGATTTACTGGGCGTAAAGCGTGCGTAGGCGGCTTTTTAAGTCGGATGTGAAATCCCTGAGCTTAACTTAGGAATTGCATTCGATACTGGAAAGCTAGAGTATGGGAGAGGATGGTAGAATTCCAGGTGTAGCGGTGAAATGCGTAGAGATCTGGAGGAATACCGATGGCGAAGGCAGCCATCTGGCCTAATACTGACGCTGAGGTACGAAAGCATGGGGAGCAAACAGGATTAGATACCCTGGTAGTCCATGCCGTAAACGATGTCTACTAGCCGTTGGGGCCTTTGAGGCTTTAGTGGCGCAGCTAACGCGATAAGTAGACCGCCTGGGGAGTACGGTCGCAAGACTAAAACTCAAATGAATTGACGGGGCCCGCACAGCGGTGGAGCATGTGGTTAATTCGATGCAACGCGAAACCTTACCTGGCCTTGAATACTAGAACTTTCCAGAAGATGATGTGCCTCGGGAACTTAGATACAGTGCTGCATGCTGTCGTAGCTCGTGTCGATGTGGTTAAGTCCCGCACACAACCTTTCTATTGCAGCGGTCATGCGGACTTTAAGAACTGCCATGACAACTGAGGAAGGCGGACACGTCAAGTCATCATGTCTTACGGCAGGTTACAC

>(GQ284530.1) Acinetobacter sp. TDSAS2-27

GGGT-ACCTTGGTACCTGACCTAGCGGCGGACGGGTGAGTAATGCTTAGGAATCTGCCTATTAGTGGGGGACAACTTCCGAAAGGAATGCTAATACCGCATACGCCTACGGGGGAAAGCAGGGGATCTTCGGACCTTGCGCTAATAGATGAGCCTAAGTCGGATTAGCTAGTTGGTGGGGTAAAGGCCTACCAAGGCGACGATCTGTAGCGGGTCTGAGAGGATGATCCGCCACACTGGGACTGAGACACGGCCCAGACTCCTACGGGAGGCAGCAGTGGGGAATATTGGACAATGGGCGCAAGCCTGATCCAGCCATGCCGCGTGTGTGAAGAAGGCCTTTTGGTTGTAAAGCACTTTAAGCGAGGAGGAGGCTCCTTTAGTTAATACCTAAAGGAGTGGACGTTACTCGCAGAATAAGCACCGGCTAACTCTGTGCCAGCAGCCGCGGTAATACAGAGGGTGCGAGCGTTAATCGGATTTACTGGGCGTAAAGCGTGCGTAGGCGGCTTTTTAAGTCGGATGTGAAATCCCTGAGCTTAACTTAGGAATTGCATTCGATACTGGAAAGCTAGAGTATGGGAGAGGATGGTAGAATTCCAGGTGTAGCGGTGAAATGCGTAGAGATCTGGAGGAATACCGATGGCGAAGGCAGCCATCTGGCCTAATACTGACGCTGAGGTACGAAAGCATGGGGAGCAAACAGGATTAGATACCCTGGTAGTCCATGCCGTAAACGATGTCTACTAGCCGTTGGGGCCTTTGAGGCTTTAGTGGCGCAGCTAACGCGATAAGTAGACCGCCTGGGGAGTACGGTCGCAAGACTAAAACTCAAATGAATTGACGGGGCCCGCACAGCGGTGGAGCATGTGGTTAATTCGATGCAACGCGAAACCTTACCTGGCCTTGAATACTAGAACTTTCCAGA-GATGATGTGCCTCGGGAACTTAGATACAGTGCTGCATGCTGTCGTAGCTCGTGTCGATGTGGTTAAGTCCCGCACACAACCTTTCTATTGCAGCGGTAATGCGGACTTTAAGAACTGCCATGACAACTGAGGAAGGCGGACACGTCAAGTCATCATGGCTTACGGCAGGGTACAC

>lcl|Query_41350 SO3

GCATATACATGCAAGTCGAGCGGATTGAAGGGAGCTTGCTCCCTGATATTAGCGGCGGACGGGTGAGTAACACGTGGGCAACCTGCCCTGCAGATGGGGATAACTCCGGGAAACCGGGGCTAATACCGAATAATCGGTTCTTCCGCATGGAAGAACTCTGAAAGACGGTTTCGGCTGTCACTGCAGGATGGGCCCGCGGCGCATTAGCTAGTTGGTGGGGTAACGGCCTACCAAGGCGACGATGCGTAGCCGACCTGAGAGGGTGATCGGCCACACTGGGACTGAGACACGGCCCAGACTCCTACGGGAGGCAGCAGTAGGGAATCTTCCACAATGGACGAAAGTCTGATGGAGCAACGCCGCGTGAGCGAAGAAGGTTTTCGGATCGTAAAGCTCTGTTGCGAGGGAAGAACAAGTACGGGAGTAACTGCCCGTACCTTGACGGTACCTCGTTAGAAAGCCACGGCTAACTACGTGCCAGCAGCCGCGGTAATACGTAGGTGGCAAGCGTTGTCCGGAATTATTGGGCGTAAAGCGCGCGCAGACGGTCCTTTAAGTCTGATGTGAAAGCCCACGGCTCAACCGTGGAGGGTCATTGGAAACTGGAGGACTTGAGTACAGAAGAGGAAAGTGGAATTCCACGTGTAGCGGTGAAATGCGTAGAGATGTGGAGGAACACCAGTGGCGAAGGCGACTTTCTGGTCTGTAACTGACGTTGAGGCGCGAAAGCGTGGGGAGCAAACAGGATTAGATACCCTGGTAGTCCACGCCGTAAACGATGAGTGCTAAGTGTTAGGGGTTTCCGCCCCTTAGTGCTGCAGCTAACGCATTAAGCACTCCGCCTGGGGAGTACGGCCGCAAGGCTGAAACTCAAAGGAATTGACGGGGACCCGCACAAGCGGTGGAGCATGTGGTTTAATTCGAAGCAACGCGAAGGACCTTACCAGTCTTGACATCCCCGCTGACCGGCATGGAACATGTCTTTCCCTTCGGGAAGCGGTGACAGGTGGTGCATGGATGTCGTCAGCTCGTGTCGGAATGGTGGTAGTCCCGCACGAGCGCAACCTTGATCTATGGCAGCATTCAGTTGGCACCTAGTGACTGCGTGAAACCGAGAGG

>(KC257005.1) Sporosarcina sp. HB31

GCCTATAC-TGCGAGTCGAGCGGATTGATGGGAGCTTGCTCCCTGATATCAGCGGCGGACGGGTGAGTAACACGTGGGCAACCTGCCCTGCAGATGGGGATAACTCCGGGAAACCGGGGCTAATACCGAATAATCGGTTCTTCCGCATGGAAGAACTCTGAAAGACGGTTTCGGCTGTCACTGCAGGATGGGCCCGCGGCGCATTAGCTAGTTGGTGGGGTAATGGCCTACCAAGGCGACGATGCGTAGCCGACCTGAGAGGGTGATCGGCCACACTGGGACTGAGACACGGCCCAGACTCCTACGGGAGGCAGCAGTAGGGAATCTTCCACAATGGACGAAAGTCTGATGGAGCAACGCCGCGTGAGCGAAGAAGGTTTTCGGATCGTAAAGCTCTGTTGCGAGGGAAGAACAAGTACGGGAGTAACTGCCCGTACCATGACGGTACCTCGTCAGAAAGCCACGGCTAACTACGTGCCAGCAGCCGCGGTAATACGTAGGTGGCAAGCGTTGTCCGGAATTATTGGGCGTAAAGCGCGCGCAGGCGGTCCTTTAAGTCTGATGTGAAAGCCCACGGCTCAACCGTGGAGGGTCATTGGAAACTGGAGGACTTGAGTACAGAAGAGGAAAGCGGAATTCCACGTGTAGCGGTGAAATGCGTAGAGATGTGGAGGAACACCAGTGGCGAAGGCGGCTTTCTGGTCTGTAACTGACGCTGAGGCGCGAAAGCGTGGGGAGCAAACAGGATTAGATACCCTGGTAGTCCACGCCGTAAACGATGAGTGCTAAGTGTTAGGGGTTTCCGCCCCTTAGTGCTGCAGCTAACGCATTAAGCACTCCGCCTGGGGAGTACGGCCGCAAGGCTGAAACTCAAAGGAATTGACGGGGACCCGCACAAGCGGTGGAGCATGTGGTTTAATTCGAAGCAACGCGAAGAACCTTACCAGTCTTGACAT-CCCGCTGACCGGCATGGAACATGTCTTTCCCTTCGGGAAGCGGTGACAGGTGGTGCATGGTTGTCGTCAGCTCGTGTCGGAATGTTGGTAGTCCCGCACGAGCGCAACCTTGATCTATTGCAGCATTCAGTTGGCACCTAGTGACTGCGTGAAACCG-GAGG

>lcl|Query_78584 PI2

GGCAGGATACAATGCAGTCGTAACAAGGTAACCGAGGTTGCTGCTTTTTGACTATTGGGGACGGGGAGTAATGCTGGGAAACTGCCTGATGGAGGGGGATAATTACTGGAAACGGCGTATACCGCCTAACGCCTTTACCACACATGGGGACCTTCGCCTCTTGCCTTCGGATGGCCCTATGGGATTACCTAGAGTGGGGTAACGGTCACCTAGGACATCCCTGTGGCTGAAGGATGACCAGCCACACTGGAACTGAACTGGGTCCAGACTCCTACGGGAGGTCCTATGGGGAATATTGCACAATGGGCGCAAGCCTGATGCACCATGCCGGGTGATGAACAAGGCCTTCGGGTTGGATTTTACTTTCACGGTATGAAGGGACAGGGATAATAACCCTGCATTGGCTTACCCTTAAAGAACCACCGGTAACTCCCGCCTACTCCGCGCAATACGAGGTGCAACCGTTAATCAATTACTGGGCGTATATTCACGCAGGGCTGTCAAGTCGATGTAAATCCCCGGTCAACCTGGAACTGCTTCGAAACTGTTGCTAATGCTTGATAGGGGTAGAATTCCTAGTGTAATCGAGATGGAGAGATCTGAGAATACCGTGGCGACAGCGCCCCTGGACAACTACTGACGCTCAGGCGAAATCGTGGGGAGAAACAGGATTAGATACCCTGTTAGACACCCGTAAACATGTCGACTGAGTGTGCTTGATGGGGCTTCCGG

>(MF993511.1) Enterobacter hormaechei

GGCA-GCTACAATGCAGTCGTAAC-AGG-AAGC-AGCTTGCTGCTTGCTGACGAGTGGGGACGGGGAGTAATGCTGGGAAACTGCCTGATGGAGGGGGATAACTACTGGAAACGGAGTATACCGCATAACGCCAAGACCAAAGAGGGGGACCTTCGCCTCTTGCCATCGGATGGCCCGATGGGATTAGCTAGAGTGGGGTAACGGTCATCTAGGACATCCCTGTGGCTGAAGGATGACCAGCCACACTGGAACTGAACACGGTCCAGACTCCTACGGGAGGCAGCATGGGGAATATTGCACAATGGGCGCAAGCCTGATGCACCATGCCGCGTGATGAAGAAGGCCTTCGGGTTGTAAAGTACTTTCACGGGAGGAAGGGACAGGGTTAATAACCCTGCATTGACTTACCCGCAAAGAAGCACCGGTAACTCCGGCCAGCACCGCGGAATACGAGGTGCAAGCGTTAATCAATTACTGGGCGTAAAGCCACGCAGGGCTGTCAAGTCGATGTAAATCCCCGGTCAACCTGGAACTGCTTCGAAACTGGCGCTAAGTCTTGAGAGGGGTAGAATTCCAGGTGTAGCGGTGATGGAGAGATCTGAGAATACCGTGGCGAAGGCGCCCCTGGACAAAGACTGACGCTCAGGCGAAAGCGTGGGGAGAAACAGGATTAGATACCCTGGTAGTCACCCGTAAACATGTCGACTGAGTGTGCTTGAGGGGGCTTCCGG

>lcl|Query_104344 SO1

CGGGGCTAATACATGCAGTCGAGCGAACTGATTAGAAGCTTGCTTCTATGACGTTAGCGGCGGACGGGTGAGTAACACGTGGGCAACCTGCCTGTAAGACTGGGATAACTTCGGGAAACCGAAGCTAATACCGGATAGGATCTTCTCCTTCATGGGAGATGATTGAAAGATGGTTTCGGCTATCACTTACAGATGGGCCCGCGGTGCATTAGCTAGTTGGTGAGGTAACGGCTCACCAAGGCAACGATGCATAGCCGACCTGAGAGGGTGATCGGCCACACTGGGACTGAGACACGGCCCAGACTCCTACGGGAGGCAGCAGTAGGGAATCTTCCGCAATGGACGAAAGTCTGACGGAGCAACGCCGCGTGAGTGATGAAGGCTTTCGGGTCGTAAAACTCTGTTGTTAGGGAAGAACAAGTACAGAGTAACTGCTGTACCTTGACGGTACCTAACCAGAAAGCCACGGCTAACTACGTGCCAGCAGCCGCGGTAATACGTAGGTGGCAAGCGTTATCCGGAATTATTGGGCGTAAAGCGCGCGCAGGCGGTTTCTTAAGTCTGATGTGAAAGCCCACGGCTCAACCGTGGAGGGTCATTGGAAACTGGGGAACTTGAGTGCAGAAGAGAAAAGCGGAATTCCACGTGTAGCGGTGAAATGCGTAGAGATGTGGAGGAACACCAGTGGCGAAGGCGGCTTTTTGGTCTGTAACTGACGCTGAGGCGCGAAAGCGTGGGGAGCAAACAGGATTAGATACCCTGGTAGTCCACGCCGTAAACGATGAGTGCTAAGTGTTAGAGGGTTTCCGCCCTTTAGTGCTGCAGCTAACGCATTAAGCACTCCGCCTGGGGAGTACGGTCGCAAGACTGAAACTCAAAGGAATTGACGGGGCCCGCACAAGCGGTGAGCATGTGGTTAATTCGAAGCAACGCGAAGAACCTTACCAGTCTGACATCCTCTGACAACTCTAAATAGAGCGTCCCTCGGGGACAAGTGACAGTGTGCATGATGTCGTCAGCCGTCTGAAATGTGGGTAGTCCCGCACGAGCGCACCCTGATCTAATGCAGCATCAGTGCACTCTAGACTGCGGTGACACGAGAAGGTGGGATGACTCATCCATGCCTTAGACTGGCTAACTGCTCAATGATGTCACAAGCTGCAGAAA

>(MK629803.1) Priestia megaterium

CGGGGCT-ATACATGCAGTCGAGCGAACTGATTAGAAGCTTGCTTCTATGACGTTAGCGGCGGACGGGTGAGTAACACGTGGGCAACCTGCCTGTAAGACTGGGATAACTTCGGGAAACCGAAGCTAATACCGGATAGGATCTTCTCCTTCATGGGAGATGATTGAAAGATGGTTTCGGCTATCACTTACAGATGGGCCCGCGGTGCATTAGCTAGTTGGTGAGGTAACGGCTCACCAAGGCAACGATGCATAGCCGACCTGAGAGGGTGATCGGCCACACTGGGACTGAGACACGGCCCAGACTCCTACGGGAGGCAGCAGTAGGGAATCTTCCGCAATGGACGAAAGTCTGACGGAGCAACGCCGCGTGAGTGATGAAGGCTTTCGGGTCGTAAAACTCTGTTGTTAGGGAAGAACAAGTACAGAGTAACTGCTGTACCTTGACGGTACCTAACCAGAAAGCCACGGCTAACTACGTGCCAGCAGCCGCGGTAATACGTAGGTGGCAAGCGTTATCCGGAATTATTGGGCGTAAAGCGCGCGCAGGCGGTTTCTTAAGTCTGATGTGAAAGCCCACGGCTCAACCGTGGAGGGTCATTGGAAACTGGGGAACTTGAGTGCAGAAGAGAAAAGCGGAATTCCACGTGTAGCGGTGAAATGCGTAGAGATGTGGAGGAACACCAGTGGCGAAGGCGGCTTTTTGGTCTGTAACTGACGCTGAGGCGCGAAAGCGTGGGGAGCAAACAGGATTAGATACCCTGGTAGTCCACGCCGTAAACGATGAGTGCTAAGTGTTAGAGGGTTTCCGCCCTTTAGTGCTGCAGCTAACGCATTAAGCACTCCGCCTGGGGAGTACGGTCGCAAGACTGAAACTCAAAGGAATTGACGGGGCCCGCACAAGCGGTGAGCATGTGGTTAATTCGAAGCAACGCGAAGA-CCTTACCAGTCTGACATC-TCTGACA-CTCTAAATAGAGCGTCCCTCGGGGACAAGTGACAGTGTGCATGATGTCGTCAGCCGTCTGAGATGTGGGTAGT-CCGCACGAGCGCACCCTGATCTAGTGCAGCATCAGTGCACTCTAGACTGCG-TGACACGAGA-G-TGG-ATGACTCATCCATGCCTTAGACT-----------------------------------
